# Fibrotic Remodeling in Retinal Vascular Diseases: Cellular Origins, Molecular Mechanisms, and Therapeutic Perspectives

**DOI:** 10.3390/cells15191772

**Published:** 2026-09-29

**Authors:** Jia Yu Ning, Brian Wang, Miyuki Ishida Yamamoto, Kaori H. Yamada

**Affiliations:** 1 Department of Pharmacology and Regenerative Medicine, University of Illinois College of Medicine, Chicago, IL 60612, USA; jning6@uic.edu (J.Y.N.); bzwang3@uic.edu (B.W.); miyukiy@uic.edu (M.I.Y.); 2 Department of Ophthalmology and Visual Sciences, University of Illinois College of Medicine, Chicago, IL 60612, USA

**Keywords:** fibrosis, neovascular age-related macular degeneration, proliferative diabetic retinopathy

## Abstract

**Highlights:**

**What are the main findings?**

- This review summarizes the cellular and molecular mechanisms of ocular fibrosis.
- This review also summarizes current therapies and their limitations, as well as potential treatments in pre-clinical studies and clinical trials.

**What are the implications of the main findings?**

- Understanding the molecular basis of ocular fibrosis may facilitate the identification of novel therapeutic targets.
- The limitations of current therapies underscore the need for more effective and targeted interventions for ocular fibrosis.

**Abstract:**

Fibrosis is a major vision-threatening complication of neovascular retinal diseases, including neovascular age-related macular degeneration (nAMD) and proliferative diabetic retinopathy (PDR). Although vascular endothelial growth factor (VEGF) inhibition effectively suppresses vascular leakage and neovascularization, some patients still develop fibrosis and experience irreversible vision loss. Recent studies have identified multiple interconnected signaling pathways that contribute to fibrotic progression, highlighting the potential need for combination or multitargeted therapeutic strategies. This review summarizes the cellular origins of myofibroblasts, the molecular mechanisms underlying fibrosis, and recent advances in antifibrotic therapies. We particularly highlight the angio-fibrotic switch, mesenchymal transition, cellular stress responses, extracellular matrix remodeling, mechanotransduction, and emerging therapeutic approaches for ocular fibrosis. Relevant preclinical and clinical studies were identified through searches of PubMed and clinical trial registries.

## 1. Introduction

Fibrosis is a major vision-threatening complication of neovascular retinal diseases, including nAMD and PDR. DR is a leading cause of vision impairment and blindness among working-age adults [1]. In contrast, nAMD is a leading cause of severe vision impairment among older adults, particularly in high-income countries [2].

**Figure 1:**
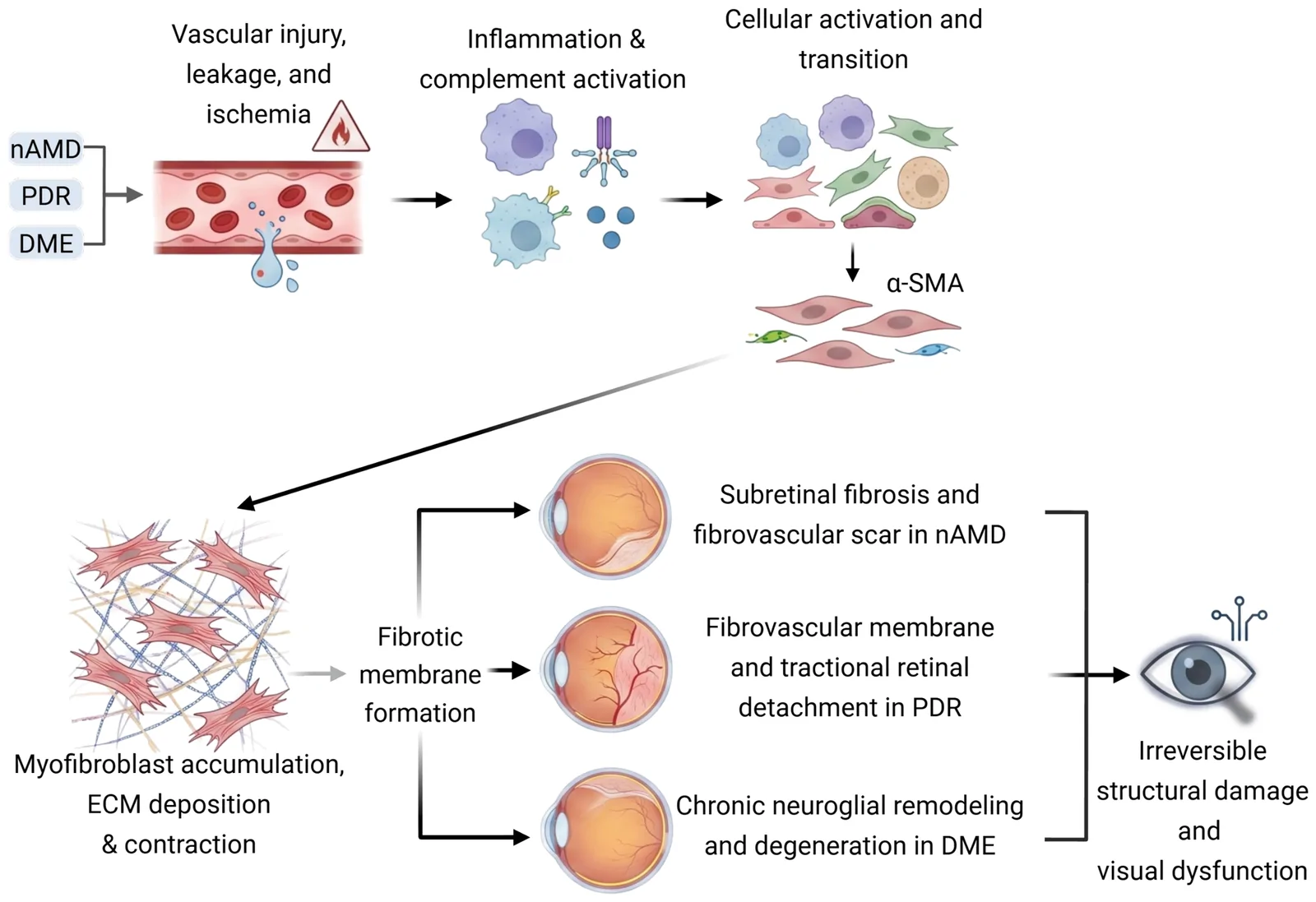
Common mechanisms contributing to fibrotic remodeling in retinal vascular diseases. Vascular injury, leakage, and ischemia associated with neovascular age-related macular degeneration (nAMD), proliferative diabetic retinopathy (PDR), and diabetic macular edema (DME) promote inflammation, complement activation, and recruitment of inflammatory cells. These responses activate resident mesenchymal cells and induce mesenchymal and profibrotic transitions in multiple cell populations, leading to myofibroblast accumulation, extracellular matrix deposition, and tissue contraction. The resulting remodeling manifests differently across retinal diseases, including subretinal fibrosis and fibrovascular scarring in nAMD, fibrovascular membrane formation and tractional retinal detachment in PDR, and chronic neuroglial remodeling and degeneration in DME. These structural changes may ultimately cause irreversible tissue damage and visual dysfunction. Abbreviations: DME, diabetic macular edema; ECM, extracellular matrix; nAMD, neovascular age-related macular degeneration; PDR, proliferative diabetic retinopathy. Created in BioRender. Yamada, K. (2026) https://BioRender.com/v5ucajx.

In nAMD, fibrosis typically develops in the subretinal space secondary to macular neovascularization (MNV) [3,4]. In contrast, in PDR, pathological retinal neovascularization is accompanied by the formation of fibrovascular membranes (FVMs) at the vitreoretinal interface [5,6,7]. Progressive extracellular matrix (ECM) deposition and contraction of these membranes can exert traction on the retina, resulting in tractional retinal detachment and irreversible visual impairment (Figure 1) [5].

Despite these anatomical differences, nAMD and PDR share several pathogenic features, including abnormal angiogenesis, chronic inflammation, mesenchymal activation, myofibroblast accumulation, and excessive ECM remodeling. This review summarizes the cellular and molecular mechanisms underlying the transition from angiogenesis to fibrosis in neovascular retinal diseases, with particular emphasis on mesenchymal transition, profibrotic signaling pathways, and current and emerging clinical management strategies.

## 2. Clinical and Pathological Overview of Fibrosis in nAMD and DR

### 2.1. Fibrosis in Neovascular AMD

In nAMD, fibrosis develops in association with MNV and can involve the tissue compartment beneath or above the retinal pigment epithelium (RPE), ultimately leading to irreversible vision loss [8,9]. MNV, characterized by the growth of abnormal blood vessels within the macula, is driven by elevated levels of vascular endothelial growth factor A (VEGF-A) and can cause exudation, hemorrhage, and retinal damage [10,11,12,13]. Accordingly, anti-VEGF therapies effectively suppress MNV and associated vascular leakage in patients with nAMD [14,15,16]. Because subretinal fibrosis develops secondary to MNV [3,4], its incidence among patients with nAMD has declined since the introduction of anti-VEGF therapies in 2006 [4]. However, anti-VEGF therapies do not directly prevent the development of subretinal fibrosis [17]. Approximately 45% of nAMD patients develop subretinal fibrosis despite ongoing anti-VEGF therapy [3], and fibrosis is the strongest predictor of poor visual prognosis [8].

Although neovascular lesions in nAMD were traditionally considered to originate from the choroidal circulation and termed choroidal neovascularization (CNV), some lesions arise from the retinal circulation. Therefore, the broader term macular neovascularization (MNV) is now preferred, with CNV representing a subset of MNV [18]. MNV is classified according to the anatomical location and presumed origin of the neovascular complex: type 1 MNV, formerly termed occult CNV, originates from the choroid and grows beneath the RPE; type 2 MNV, formerly termed classic CNV, extends through the RPE into the subretinal space; and type 3 MNV, previously termed retinal angiomatous proliferation, originates within the retina and extends toward the outer retina and RPE, often accompanied by intraretinal edema, hemorrhage, and telangiectatic changes [18]. Predominantly classic CNV, which broadly corresponds to type 2 MNV in the current nomenclature, is associated with an increased risk of fibrotic scar formation [3,19].

Clinically, the presence of subretinal hyperreflective material (SHRM) on optical coherence tomography (OCT) has been identified as a risk factor for fibrosis. Eyes showing a reduction in SHRM thickness during anti-VEGF therapy have a better visual prognosis than those with treatment-resistant SHRM [20,21]. Detailed studies using spectral-domain OCT have further demonstrated the presence of SHRM during the progression of subretinal fibrosis in eyes with nAMD [22]. Longitudinal analysis identified three distinct pathways of fibrosis progression originating from CNV: (1) progression to fibrosis beneath the RPE followed by RPE erosion, the appearance of SHRM, and the development of fibrosis above the RPE, ultimately rendering the residual RPE indistinguishable; (2) progression of fibrosis above the RPE, followed by loss of a distinguishable RPE layer, and (3) persistence of fibrosis beneath the RPE, accompanied by the development of a flat, fibroatrophic lesion [22]. Subretinal fibrosis encompasses a spectrum of fibrotic lesions associated with nAMD, ranging from subtle fibrosis detectable by OCT to large, clinically apparent disciform scars representing an advanced stage of the disease [9,23].

### 2.2. Fibrosis in Diabetic Retinopathy

Diabetic vascular complications are major contributors to morbidity and mortality and affect multiple organs, including the kidneys (diabetic nephropathy), eyes (diabetic retinopathy, DR), and peripheral nerves (diabetic neuropathy) [24]. DR is the most common microvascular complication of diabetes [24,25]. Based on the extent of microvascular degeneration and associated ischemic damage, DR is broadly classified into two stages: non-proliferative diabetic retinopathy (NPDR) and the more advanced stage, proliferative diabetic retinopathy (PDR) [24].

The development and progression of DR involve multiple vascular abnormalities, including breakdown of the blood–retina barrier (BRB), progressive microvascular damage characterized by the loss of vascular endothelial cells and pericytes, capillary occlusion, and thickening of the vascular basement membrane (BM). DR is also accompanied by neuronal and glial abnormalities in the retina [24]. When vascular leakage affects the macula and causes retinal thickening or swelling, the condition is termed diabetic macular edema (DME) [24]. PDR and DME represent the two major sight-threatening manifestations of diabetic eye disease [24].

PDR is characterized by pathological retinal neovascularization and formation of epiretinal FVM at the vitreoretinal interface (Figure 1) [25]. Excessive VEGF-A signaling promotes aberrant blood vessel growth from the retina into the vitreous, contributing to FVM formation. Subsequent contraction of FVMs can exert traction on the retina, leading to tractional retinal detachment. In advanced cases, surgical removal of the FVMs may be required to relieve vitreoretinal traction and prevent or treat retinal detachment [5].

In DME, breakdown of the BRB leads to fluid accumulation in the macula [26]. Extracellular fluid may accumulate within the retinal tissue, forming a cavity-like structure known as a cyst, or in the subretinal area, forming subretinal fluid (SRF). Visual impairment may be reversible following fluid resolution; however, persistent or recurrent edema can disrupt the normal architecture of the macula and lead to irreversible vision loss [27,28]. Fluid may also accumulate intracellularly, causing cell swelling. Prolonged swelling and degeneration of Müller cells and adjacent neurons have been proposed to contribute to the formation of cystoid spaces in chronic DME [26]. Because Müller cells play essential roles in retinal fluid clearance, neuronal homeostasis, and maintenance of BRB integrity, their dysfunction can further exacerbate fluid accumulation, inflammatory cytokine production, and loss of neuronal support, thereby promoting persistent neuroglial remodeling [26].

Several OCT features, including disorganization of the retinal inner layers (DRIL), disruption of the external limiting membrane (ELM), ellipsoid zone (EZ), and cone outer segment termination (COST) line, as well as outer retinal thinning, are associated with poorer visual acuity in DME [28]. Although anti-VEGF therapy can effectively reduce vascular leakage and retinal thickening, the resolution of macular edema does not necessarily restore normal retinal architecture. Prolonged or recurrent edema may induce persistent neuroglial remodeling, including Müller cell gliosis, disorganization of the retinal layers, photoreceptor damage, neuronal loss, and retinal thinning, thereby limiting visual recovery despite anatomical improvement.

These chronic structural changes in DME are more appropriately characterized as neuroglial remodeling and degeneration than as overt fibrosis. Nevertheless, diabetes induces profibrotic remodeling of the retinal extracellular matrix, including excessive deposition of fibronectin and other matrix proteins in the retinal capillary basement membrane. This fibrotic subendothelial matrix may compromise endothelial barrier integrity and contribute to persistent vascular leakage. Thus, although DME does not typically produce a well-defined fibrotic scar comparable to subretinal fibrosis in nAMD or preretinal fibrovascular membranes in PDR, chronic edema develops within a profibrotic retinal microenvironment and may, in rare cases, be accompanied by intraretinal fibrosis [29].

## 3. Cellular Origins and Mesenchymal Transition in Ocular Fibrosis

### 3.1. Mesenchymal Transition in Fibrotic Remodeling

Myofibroblasts play an important role in normal wound healing, during which they are transiently activated to deposit ECM proteins such as collagen and fibronectin, and promote wound contraction. Additionally, myofibroblasts contain actin–myosin bundles containing α-smooth muscle actin (α-SMA), which aid contraction of granulation tissue [30,31]. Although abundant in retinal and subretinal fibrotic lesions, myofibroblasts are not typically present in healthy macular tissue. Therefore, the myofibroblast population within these lesions may be derived from circulating precursors or transdifferentiated from various resident cell types.

#### 3.1.1. EMT and EndMT

**Figure 2:**
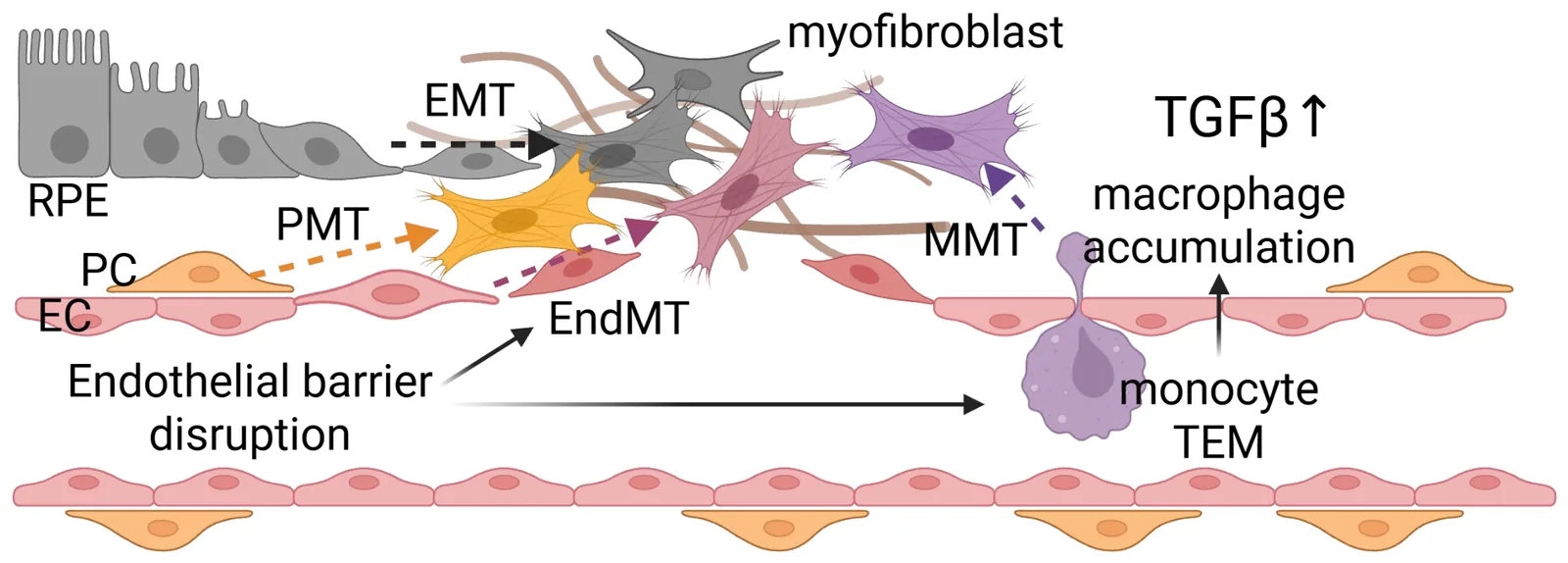
Potential cellular origins of myofibroblasts during fibrotic remodeling. Myofibroblasts may arise from multiple cellular sources, including resident fibroblasts; epithelial cells undergoing epithelial-to-mesenchymal transition; endothelial cells undergoing endothelial-to-mesenchymal transition; pericytes and other perivascular mesenchymal cells; and recruited circulating or bone marrow-derived cells. These distinct cell populations may acquire overlapping mesenchymal and profibrotic properties, characterized by increased migration, contractility, and extracellular matrix production. The relative contribution of each cellular source varies according to the affected tissue, disease context, and stage of fibrosis. Abbreviations: RPE, retinal pigment epithelium; EC, endothelial cell; PC, pericyte; EMT, epithelial-to-mesenchymal transition; EndMT, endothelial-to-mesenchymal transition; PMT, pericyte-to-myofibroblast transition; TEM, transendothelial migration; MMT, macrophage-to-myofibroblast transition; TGF-β, transforming growth factor-β. Created in BioRender. Yamada, K. (2026) https://BioRender.com/cpirnno.

Evidence supports epithelial and endothelial cells as sources of myofibroblasts through epithelial-to-mesenchymal transition (EMT) and endothelial-to-mesenchymal transition (EndMT), respectively (Figure 2). While these processes are essential in embryonic implantation and development, dysregulated EMT and EndMT contribute to pathological fibrosis [32]. In fibrosis secondary to nAMD and DR, RPE [4,33,34] and choroidal endothelial cells (CECs) [34] undergo transdifferentiation into myofibroblasts in response to pro-fibrotic growth factors and inflammatory cytokines, most notably transforming growth factor beta (TGF-β) [32,34,35,36].

During EMT and EndMT, epithelial and endothelial cells lose their apical-basal polarity and junctional proteins and acquire a highly migratory and invasive phenotype [37]. EMT is characterized by a downregulation of epithelial markers such as E-cadherin and Zonula Occludens-1 (ZO-1) with a concomitant upregulation of mesenchymal markers such as N-cadherin, α-SMA, vimentin, fibronectin, and collagen-1 [33,34,38]. EndMT follows a similar pattern, with a loss of endothelial markers such as VE-cadherin, CD31, and endothelial nitric oxide synthase (eNOS) and acquisition of mesenchymal markers [34,36].

**Figure 3:**
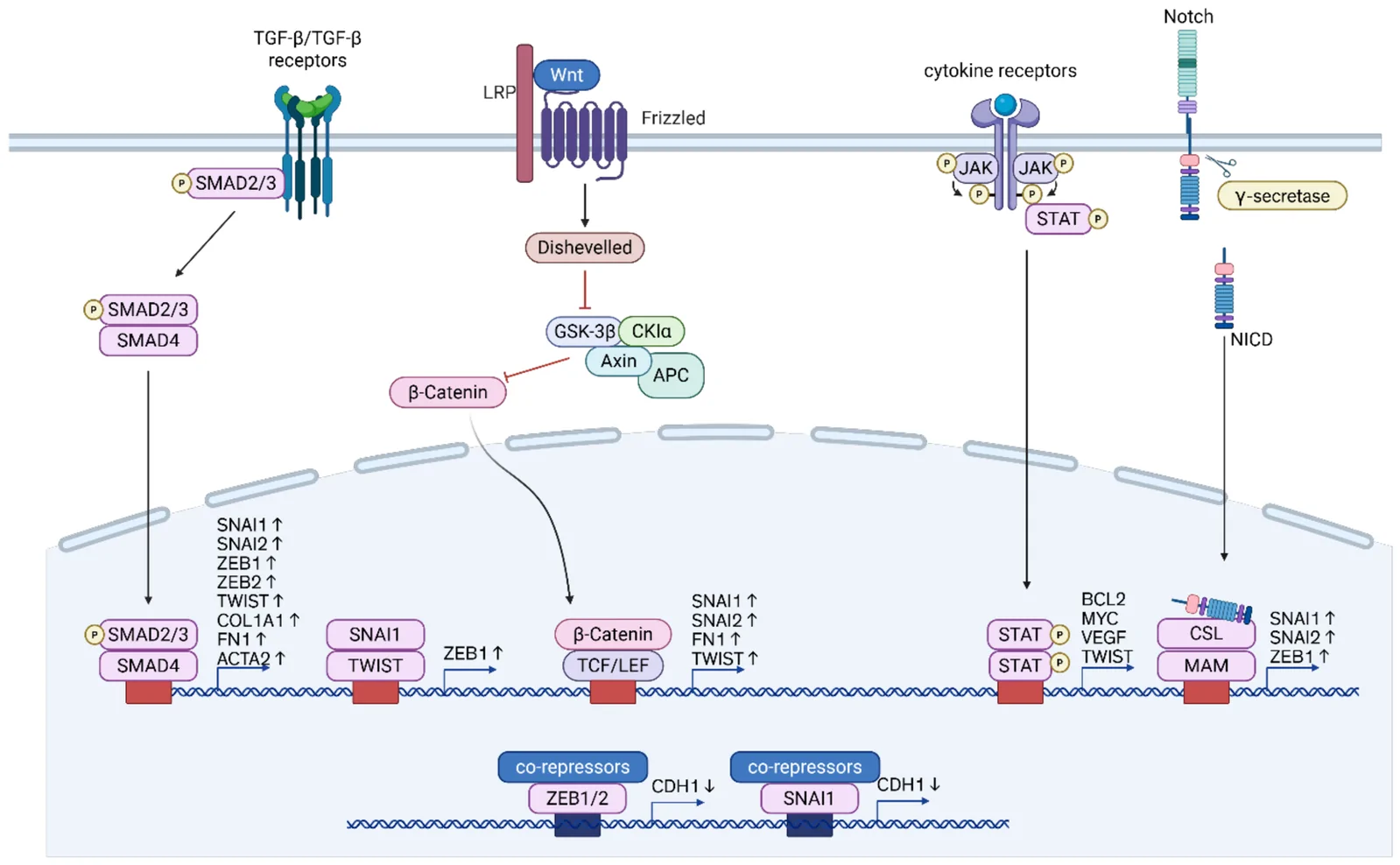
Signaling pathways regulating mesenchymal transition during fibrotic remodeling. TGF-β/SMAD, Wnt/β-catenin, JAK/STAT, and Notch signaling converge on transcriptional regulators, including SNAI1/2, ZEB1/2, and TWIST, to promote mesenchymal and profibrotic gene expression while repressing epithelial or cell-type-specific identity genes such as CDH1. Although these mechanisms are commonly described in the context of epithelial-to-mesenchymal transition (EMT), related transcriptional programs can be activated in multiple epithelial and non-epithelial cell populations. Such cell-type-specific mesenchymal transitions may collectively promote extracellular matrix production, cellular migration, contractility, and tissue remodeling during ocular fibrosis. Black arrows indicate translocation of the molecules. Blue arrows indicate gene expression. Red line indicate inhibition of the molecules. Created in BioRender. Yamada, K. (2026) https://BioRender.com/y247gps.

Key transcription factors, such as Snail (SNAI1), Slug (SNAI2), zinc finger E-box binding homeobox (ZEB) 1/2, and Twist1/2, suppress epithelial and endothelial adhesion proteins (e.g., E-cadherin, VE-cadherin) while activating mesenchymal gene expression (Figure 3) [33,39,40]. In response to TGF-β, upregulated SNAI1 cooperates with TWIST to induce expression of ZEB1 [41]. Overexpression of SNAI1 in human umbilical vein endothelial cells (HUVECs) enhanced cell motility and invasion, alongside inducing a morphological change from cobblestone-like to fibroblastic and spindle-like, whereas knockdown of SNAI1 downregulated cytoskeletal remodeling and ECM gene expression [42].

EMT-associated transcription factors, including SNAI1 and ZEB1/2, suppress E-cadherin expression by binding to E-box elements in the *CDH1* (E-cadherin gene) promoter and recruiting chromatin-modifying corepressor complexes (Figure 3) [43]. Members of the miR-200 family, which are downregulated during EMT, counteract this process by directly targeting ZEB1/2, thereby restoring E-cadherin expression [44]. In the diabetic retina, miR-200b has also been implicated in regulating EndMT. High-glucose conditions reduce miR-200b expression in retinal endothelial cells and induce the loss of endothelial markers and the acquisition of mesenchymal markers through TGF-β-dependent signaling. Conversely, endothelial-specific overexpression of miR-200b prevents these phenotypic changes in diabetic mice, suggesting that loss of miR-200b may contribute to diabetes-induced retinal EndMT and extracellular matrix accumulation [45]. Like Snail and ZEB, expression of the helix–loop–helix (HLH) protein TWIST downregulates epithelial markers and increases expression of the mesenchymal markers, vimentin and N-cadherin [46].

#### 3.1.2. Differentiation of Circulating Bone Marrow-Derived Fibrocytes

Fibrocytes are matrix-producing cells circulating in the peripheral blood that express hematopoietic stem cell markers (e.g., CD34) and mesenchymal markers (e.g., collagen-1, vimentin, fibronectin) [47,48,49]. Upon recruitment to tissue injury sites, the CD34 marker is downregulated during wound repair as fibrocytes differentiate into mature mesenchymal cells, such as fibroblasts and myofibroblasts [48]. This transition is driven by TGF-β, which also regulates EndMT, EMT, and fibrosis. Stimulation of peripheral blood mononuclear cells (PBMCs) with TGF-β promoted fibrocyte differentiation in vitro. TGF-β promoted fibrocyte differentiation, and the differentiated cells expressed α-SMA and contracted collagen gels, consistent with a myofibroblast-like phenotype [50].

In a two-stage laser-induced mouse model of subretinal fibrosis secondary to CNV, circulating CD45^+^/collagen I^+^ fibrocytes increased following fibrosis induction and were detected within fibrotic lesions; moreover, aged mice exhibited higher fibrocyte numbers and more extensive fibrosis than young mice [51]. In human ocular fibrosis, cells co-expressing hematopoietic or fibrocyte-associated markers and α-SMA have been identified in epiretinal membranes from patients with proliferative vitreoretinopathy, suggesting that circulating fibrocytes may contribute to the myofibroblast population [52]. However, their direct contribution to subretinal fibrosis in patients with nAMD remains to be established.

#### 3.1.3. Pericyte-to-Myofibroblast Transition (PMT)

Pericytes, specialized mural cells that stabilize microvessels, regulate blood flow, and vascular homeostasis [53], represent another potential source of myofibroblasts through pericyte-to-myofibroblast transition (PMT) (Figure 2) [54]. Given the abundance of pericytes in retinal vessels, damage to pericytes destabilizes microvasculature and promotes neovascularization [55]. In the animal model of nAMD, following laser-induced photocoagulation, infiltrating choroidal pericytes expressing α-SMA were detected in regions rich in fibronectin and collagen-1, accompanied by upregulation of ECM-related transcripts [54].

Single-cell transcriptomic analysis of fibrovascular membranes from patients with PDR identified adipocyte enhancer-binding protein 1 (*AEBP1*) as a candidate regulator of PMT. AEBP1 was enriched in myofibroblast populations, and its silencing reduced the expression of profibrotic markers in human retinal pericytes exposed to high glucose. However, its therapeutic efficacy has not yet been evaluated in vivo [56].

Taken together, the loss of pericytes through PMT contributes to sustained inflammation and serves as an additional source of ECM components fueling fibrosis.

### 3.2. Contributions of Macrophages to Fibrotic Remodeling

#### 3.2.1. Macrophage-to-Myofibroblast Transition (MMT)

Beyond resident cell transdifferentiation, infiltrating macrophages are another source of myofibroblasts. In surgically excised CNV lesions from human patients, macrophages were found in 61% of specimens [57]. Similarly, fibrovascular epiretinal membranes from PDR patients contained mononuclear cells derived from monocytes/macrophages, with a subset expressing both α-SMA and fibroblast-specific protein-1 (FSP-1), consistent with macrophage-to-myofibroblast transition (MMT) [36].

Evidence from a murine dual-laser model of subretinal fibrosis and human nAMD patients further supports MMT. Approximately 30% of infiltrating F4/80+ macrophages expressed α-SMA [58]. In vitro, treatment of bone marrow-derived macrophages (BMDMs) with TGF-β significantly upregulated mesenchymal markers α-SMA, fibronectin, and collagen-1 [58,59].

#### 3.2.2. Macrophage-Driven Fibrosis

In addition to MMT, macrophages produce various profibrotic and proinflammatory mediators, such as TGF-β, interleukin-1β (IL-1β), and tumor necrosis factor (TNF), which activate myofibroblasts [60], and their accumulation directly contributes to the development of subretinal fibrosis in nAMD [33,34,61,62,63]. In idiopathic pulmonary fibrosis, the activity of differentiated alveolar macrophages fails to terminate, resulting in persistent inflammation that drives excessive ECM deposition and fibroblast proliferation [60,64]. Prolonged TGF-β production by macrophages further drives EMT of renal tubular cells [65] and MMT in renal fibrosis [66], highlighting the contributions of macrophages in both mesenchymal transitions and fibrotic remodeling.

### 3.3. Contributions of Glial Cells to Fibrotic Remodeling

Within the retina, Müller glia and astrocytes constitute the macroglial population that supports and maintains the internal BRB. In angiogenic ocular conditions such as DR and nAMD, these glial cells also promote neovascularization by secreting the pro-angiogenic growth factor VEGF [67]. Disruption of Müller glia cells activates TGF-β signaling, which further drives pathological remodeling. Additionally, Müller glia can directly contribute to fibrotic tissue formation by producing ECM proteins, including connective tissue growth factor (CTGF) and fibronectin [68]. Retinal astrocytes may also contribute to ECM remodeling. *Cyp1b1*-deficient mouse retinal astrocytes exhibit elevated production of fibronectin and its receptors, αvβ3 and α5β1 integrins, together with enhanced cellular adhesion [69]. Glial cells contribute to fibrosis by modulating ECM remodeling and inflammatory signaling, thereby creating a permissive microenvironment for fibrotic progression.

## 4. Molecular Signaling Pathways Driving Fibrotic Remodeling

Recent studies have demonstrated that the transition toward fibrosis is regulated by a highly interconnected signaling network centered on TGF-β/SMAD signaling and involving growth factors, developmental, stress-responsive, and mechanotransduction pathways that engage in extensive crosstalk [70,71]. In the following sections, these pathways are organized into three major axes—growth factor and developmental signaling, stress-responsive and inflammatory signaling, and mechanical signaling—and their respective roles are discussed in detail.

### 4.1. The Angio-Fibrotic Switch in nAMD and PDR

In both nAMD [72] and PDR [73], angiogenic and fibrotic processes coexist within neovascular lesions. These processes are regulated in part by the balance between pro-angiogenic factors, particularly VEGF, and profibrotic mediators, such as TGF-β and CTGF/CCN2 [74]. A shift from an angiogenesis-dominant state toward a fibrosis-dominant state is commonly referred to as the “angio-fibrotic switch” [8,33]. In PDR, the vitreous CTGF-to-VEGF ratio correlates more strongly with the extent of fibrosis than either factor alone, supporting the concept that their relative abundance influences the transition from neovascularization to fibrosis. Moreover, an increase in this ratio following anti-VEGF treatment has been associated with accelerated vitreoretinal fibrosis in PDR [33].

Analysis of fibrovascular membranes from patients with PDR showed that preoperative bevacizumab treatment was associated with increased endothelin-1 expression [75]. This observation suggests that VEGF suppression may be accompanied by enhanced endothelin signaling and increased fibroblast or myofibroblast contractility during the angio-fibrotic switch.

The relationship between anti-VEGF therapy and the angio-fibrotic switch is less clearly established in nAMD. Nevertheless, VEGFR2 antagonism exacerbated fibrosis and EndMT in a mouse model of spontaneous CNV [76], suggesting that inhibition of VEGF signaling may alter the balance between angiogenic and fibrotic responses under certain experimental conditions.

### 4.2. Growth Factor/Developmental Signaling Pathways

#### 4.2.1. TGF-β Signaling

Growth factor-mediated signaling pathways represent a core regulatory module that initiates and sustains fibrotic reprogramming in nAMD. Rather than functioning as isolated linear cascades, these pathways operate as an interconnected transcriptional network that drives cellular fate transitions in response to retinal injury and chronic stress. Among these, TGF-β signaling plays a central role [4,33,34,54,77]. TGF-β is elevated in rodent nAMD models [72,76] and nAMD patients’ eyes even after anti-VEGF treatments [78].

TGF-β signaling is initiated by binding to the type II TGFBR (TGFBR-II). Next, the complex recruits other TGFBRs and forms a complex with a stoichiometry of two copies of TGFBR-I, two copies of TGFBR-II, and one copy of active TGF-β [79]. Upon ligand binding, TGF-β receptors phosphorylate SMAD2/3, which form complexes with SMAD4 and translocate to the nucleus to induce the transcription of profibrotic genes such as collagen-1 (*COL1A1*), fibronectin (*FN1*), and α-SMA (*ACTA2*) (Figure 3) [80]. This signaling axis promotes myofibroblast differentiation and ECM deposition.

In the meantime, TGFBRs can also phosphorylate other signaling proteins, such as kinases, or transmit signals via crosstalking to other pathways [79]. These signaling molecules include PI3K/AKT, MEK/ERK1/2 [81], RhoA, and JNK/p38 [82]. TGF-β activates PI3K/AKT signaling, which counteracts TGF-β-induced apoptosis and growth arrest [83] and facilitates SMAD-dependent EMT [84]. TGF-β also promotes PMT through the SMAD2/3 and Akt/mTOR pathways [85]. The TGF-β-induced ERK/MAPK pathway contributes to EMT by disrupting cell–cell adherens junctions, thereby increasing cell motility [81]. RhoA-dependent signaling pathway induces stress fiber formation [82]. TGF-β activates the JNK/p38 pathway via TGF-β-activated kinase 1 (TAK1), thereby mediating TGF-β-induced apoptosis [82].

TGF-β further reduces VEGFR2 levels in ECs, thereby reducing responsiveness to anti-VEGF therapies [76]. TGF-β is a critical factor in the induction of subretinal fibrosis, as TGF-β-neutralizing antibodies inhibit fibrosis in mice [86].

#### 4.2.2. ANG2 Signaling

Angiopoietin 2 (ANG2) is a context-dependent ligand of TIE2, an endothelial receptor tyrosine kinase, and can also signal through integrins, including α5β1, α3β1, αvβ5, and αvβ3, particularly in endothelial cells with low TIE2 expression [87,88,89]. Angiopoietin 1 (ANG1) is the principal activating ligand of TIE2 and promotes endothelial survival, vascular quiescence, and barrier stability. By contrast, ANG2 functions as a context-dependent weak agonist/antagonist of TIE2. Under pathological angiogenic conditions, ANG2 generally antagonizes ANG1–TIE2 signaling, thereby destabilizing the vasculature and increasing its responsiveness to inflammatory and angiogenic stimuli, including VEGF-A [88].

ANG2 is upregulated together with VEGF, IL-1β, and TNF-α in experimental laser-induced CNV [90]. Although ANG2 is not considered a direct fibrogenic factor, it may facilitate fibrotic remodeling by promoting vascular destabilization and leakage, inflammatory-cell recruitment, and persistent tissue injury. In a spontaneous CNV model, inhibition of ANG2, VEGF-A, or both initially reduced CNV lesion area; however, only dual ANG2/VEGF-A inhibition reduced neovascular leakage and macrophage/microglia accumulation around CNV lesions. In a retinal ischemia/reperfusion injury model, dual ANG2/VEGF-A inhibition was more effective than either monotherapy in reducing vascular leakage and neurodegeneration [91]. These findings associate ANG2 with vascular instability and inflammation but do not directly demonstrate an antifibrotic effect. Experimental CNV models suggest that combined ANG2/VEGF-A inhibition reduces collagen remodeling [92]; however, a direct causal role for ANG2 in subretinal fibrosis and the clinical antifibrotic efficacy of dual ANG2/VEGF-A inhibition remain to be established.

#### 4.2.3. PDGF Signaling

Platelet-derived growth factor (PDGF)/PDGFR signaling promotes pericyte recruitment and mesenchymal cell survival, thereby contributing to the formation and stabilization of fibrovascular lesions [93]. Following tissue injury, pericytes may dissociate from endothelial cells and acquire inflammatory and profibrotic phenotypes in response to inflammatory cytokines, such as TNF-α, IL-1β, and IFN-γ, as well as TGF-β and PDGF-BB. Activated pericytes may promote fibrosis by secreting fibroblast-activating growth factors, producing proteins involved in ECM remodeling, and depositing structural and matricellular ECM proteins [93].

The PDGF family comprises four polypeptide chains, PDGF-A, PDGF-B, PDGF-C, and PDGF-D, which assemble into five biologically active disulfide-linked dimers. PDGF-A and PDGF-B form the homodimers PDGF-AA and PDGF-BB, as well as the heterodimer PDGF-AB [94]. PDGF-C [95] and PDGF-D [96,97], which were identified later, form the homodimers PDGF-CC and PDGF-DD, respectively [94]. PDGFs act via two receptor tyrosine kinases (PDGFR-α and PDGFR-β).

Among the PDGF isoforms, PDGF-BB has been particularly implicated in neovascular ocular fibrosis, because it activates PDGFRβ-expressing pericytes and mesenchymal cells, promoting their recruitment, migration, proliferation, and survival within fibrovascular lesions [98]. However, ocular fibrosis is driven by a complex network of profibrotic mediators, with TGF-β signaling playing a central role in myofibroblast differentiation and ECM deposition.

#### 4.2.4. Wnt/β-Catenin Signaling

In addition to TGF-β signaling, several interconnected signaling pathways contribute to EMT, including the canonical Wnt/β-catenin pathway. Activation of Wnt/β-catenin signaling promotes the accumulation and nuclear translocation of β-catenin, which cooperates with T-cell factor/lymphoid enhancer-binding factor (TCF/LEF) transcription factors to activate the EMT-associated transcriptional program (Figure 3). These programs involve EMT-inducing transcription factors, including members of the SNAIL, TWIST, and ZEB families, and promote the loss of epithelial characteristics and acquisition of a mesenchymal phenotype [79,99].

In an experimental model of laser-induced CNV and subretinal fibrosis, Wnt/β-catenin signaling is activated in association with TGF-β-induced RPE transition. In contrast, pharmacological inhibition of β-catenin attenuates mesenchymal marker expression and fibrotic lesion formation [100]. Mechanistically, canonical Wnt signaling can synergize with TGF-β1-induced SMAD/ZEB1/2 signaling to promote EMT-associated transcriptional reprogramming in RPE cells [101]. Consistent with this interaction, simultaneous suppression of ERK1/2 and Wnt/β-catenin signaling attenuates TGF-β-induced RPE transition and experimental subretinal fibrosis [102].

ECM-derived signals may also regulate Wnt/β-catenin activity within fibrovascular lesions. Fibronectin, a major ECM component involved in both angiogenesis and fibrosis, and its receptor integrin α5β1 are upregulated in experimental laser-induced MNV. Fibronectin silencing reduces Wnt/β-catenin signaling and suppresses endothelial proliferation, migration, and neovascular lesion formation, suggesting that the fibronectin-integrin α5β1 axis may promote fibrovascular remodeling through Wnt/β-catenin signaling [103].

Wnt/β-catenin signaling also contributes to pathological neovascularization. Very low density lipoprotein receptor gene knockout (*Vldlr*^−/−^) mice, which are widely used as a model of subretinal neovascularization, exhibit increased expression of Wnt co-receptors, low density lipoprotein receptor-related protein 5 and 6 (LRP5/6), stabilization of β-catenin, and increased VEGF expression. Inhibition of Wnt signaling with Dickkopf-1 reduces β-catenin and VEGF levels, supporting a role for canonical Wnt signaling in subretinal neovascularization [104].

The effects of Wnt/β-catenin signaling in DR are ligand-, receptor-, and cell-type-dependent. Aberrant canonical Wnt pathway activation has been implicated in retinal inflammation, vascular leakage, and pathological neovascularization. An LRP6-blocking antibody reduced β-catenin accumulation, inflammatory and angiogenic factor expression, leukostasis, vascular leakage, and pathological neovascularization in experimental models of diabetes and ischemic retinopathy [105]. Ant signaling may also contribute to fibrotic remodeling in DR. Intravitreal delivery of an adenovirus expressing SERPINA3K reduced CTGF and fibronectin expression in the retinas of streptozotocin (STZ)-induced diabetic rats by inhibiting canonical Wnt signaling [106].

Conversely, selective activation of the Norrin/FZD4/LRP5 signaling axis can support retinal vascular homeostasis and blood–retina barrier integrity. An agonist of frizzled class receptor 4 (FZD4) activated β-catenin signaling and induced the expression of blood–retina barrier-associated genes in retinal endothelial cells. In experimental retinopathy models, FZD4 activation reduced avascular areas, pathogenic neovascularization, and VEGF-induced vascular leakage [107].

Thus, Wnt/β-catenin signaling cannot be classified as uniformly pathogenic or protective in retinal disease. Its effects depend on the activating ligand and receptor complex, the responding cell type, and the stage of disease.

#### 4.2.5. Notch Signaling

Notch signaling further contributes to pathological vascular remodeling and cell fate dysregulation within the retinal microenvironment [108]. Extensive crosstalk among the TGF-β/SMAD, Wnt/β-catenin, and Jagged/Notch pathways may promote EMT-like phenotypic changes in RPE cells [109].

In mammals, the Notch signaling system comprises four receptors, NOTCH1-4, and five canonical ligands, including Delta-like ligand 1, 3, and 4 (DLL1, DLL3, and DLL4), and Jagged 1 and 2 (JAG1 and 2). Both the receptors and ligands are transmembrane proteins, allowing Notch signaling to mediate direct communication between neighboring cells [108]. Ligand binding induces sequential proteolytic cleavage of the Notch receptors, first by an A disintegrin and metalloprotease (ADAM) and subsequently by the γ-secretase complex. This processing releases the Notch intracellular domain (NICD), which translocates to the nucleus and interacts with transcriptional regulators to induce downstream target genes, including members of the HES and HEY families (Figure 3) [108].

In human RPE cells undergoing TGF-β2-induced EMT, the expression of Jagged-1 and Notch receptors is increased, together with that of the downstream effectors HES1 and HEY1 [109,110]. Genetic knockdown of Jagged-1 or pharmacological inhibition of Notch receptor cleavage attenuates TGF-β2-induced EMT and suppresses the expression of EMT-associated transcriptional factors, including SNAI1, SNAI2, and ZEB1 [110]. TGF-β2-induced upregulation of Jagged-1 involves both canonical SMAD signaling and non-canonical PI3K/AKT and MAPK pathways [111]. Moreover, reciprocal interactions between Jagged/Notch and ERK1/2 signaling may further amplify the EMT program in RPE cells [111].

Under physiological conditions, Notch signaling supports pericyte survival and contributes to the maturation and stabilization of retinal blood vessels. Ligand-dependent activation of Notch signaling protects retinal pericytes from cell death, whereas disruption of endothelial Notch signaling results in ectopic endothelial proliferation and sprouting, defective mural cell recruitment, and sustained VEGF receptor expression [112,113]. In fibrotic conditions, however, sustained Notch activation may promote pericyte proliferation and phenotypic transition. In pulmonary fibrosis, NOTCH1 activation enhances PDGFRβ/ROCK signaling and promotes PMT [114].

Pathological activation of Notch signaling also contributes to diabetic microvascular remodeling. In DR, hyperglycemia-induced upregulation of JAG1/DLL4 activates endothelial NOTCH1 signaling and disrupts adherens junction integrity through rapid noncanonical signaling involving AKT/eNOS and SRC. This leads to VE-cadherin phosphorylation and dissociation of the VE-cadherin/β-catenin complex, thereby increasing retinal vascular permeability [115]. In human blood vessel organoids exposed to hyperglycemic and inflammatory conditions, DLL4-NOTCH3 signaling was identified as a key driver of vascular basement-membrane thickening, suggesting that dysregulation of Notch signaling also contributes to diabetes-induced ECM remodeling [116].

Collectively, these pathways form an interconnected and partially redundant profibrotic signaling network. TGF-β serves as a central inducer of myofibroblast differentiation and ECM production [4,33,34,54,77], whereas PDGF supports the recruitment, migration, proliferation, and survival of pericytes and other mesenchymal cells within fibrovascular lesions [93]. Wnt/β-catenin signaling contributes to the establishment and maintenance of mesenchymal transcriptional states [79,99], while Jagged/Notch signaling reinforces TGF-β-induced EMT-like reprogramming and may interact with PDGFRβ/ROCK signaling during pericyte phenotypic transitions [114]. Thus, these pathways act at distinct but overlapping stages of fibrotic remodeling and can converge on common cellular outcomes, including myofibroblast activation, ECM accumulation, and pathological tissue remodeling. This functional overlap may allow profibrotic remodeling to persist when a single pathway is inhibited, thereby contributing to incomplete or transient responses to single-pathway therapies.

The clinical limitations of single-pathway inhibition are illustrated by the persistence of fibrotic remodeling in a subset of patients despite effective suppression of neovascular activity by anti-VEGF therapy. Similarly, the addition of the PDGF antagonist pegpleranib to anti-VEGF therapy did not improve visual outcomes in phase III trials, as discussed in Section 5.3.2. These clinical observations do not directly establish compensatory crosstalk among all four pathways. Nevertheless, they highlight the difficulty of achieving durable clinical benefit by targeting a single component of a heterogeneous fibrovascular network. Experimental evidence of reciprocal and convergent signaling among these pathways provides a mechanistic rationale for investigating molecularly defined combination strategies.

### 4.3. Stress-Responsive and Inflammatory Signaling Pathways

#### 4.3.1. Hypoxia and HIF Signaling

Stress-responsive and inflammatory processes, including hypoxia, oxidative stress, complement activation, and inflammatory cytokine signaling, reshape the local tissue microenvironment and amplify profibrotic signaling, thereby promoting disease progression [117,118,119,120].

In AMD, age-related alterations in the composition and permeability of Bruch’s membrane, together with reduced choriocapillaris density and impaired choroidal perfusion, compromise metabolic exchange and oxygen delivery to the RPE and outer retina, creating a localized hypoxic microenvironment that may contribute to the development and progression of nAMD [121]. In DR, pericyte loss, endothelial dysfunction, leukostasis, and basement membrane thickening impair the retinal microcirculation, promoting capillary occlusion and the formation of acellular capillaries, resulting in retinal ischemia and tissue hypoxia [122]. Microaneurysm and localized perfusion deficits can be detected during early NPDR, and capillary nonperfusion may occur even at early disease stages. As the nonperfused area expands, retinal ischemia and hypoxia become more pronounced and are associated with increasing DR severity and the subsequent development of pathological neovascularization [123].

Under hypoxic conditions, hypoxia-inducible factor-1α (HIF-1α) is stabilized and translocates to the nucleus, where it activates hypoxia-responsive transcriptional programs that increase the expression of VEGF, PDGF, CTGF, and other angiogenic and profibrotic mediators [124,125]. These mediators act on RPE cells, pericytes, macrophages, and glial cells, thereby promoting angiogenesis, inflammation, mesenchymal cell activation, and ECM remodeling within the fibrovascular microenvironment. Thus, persistent hypoxia helps couple pathological neovascularization to subsequent fibrotic remodeling in nAMD and DR.

#### 4.3.2. Oxidative Stress

Although oxygen availability is reduced under hypoxic conditions, impaired mitochondrial electron transport can increase electron leakage and promote the partial reduction of residual oxygen to reactive oxygen species (ROS), particularly at mitochondrial complex III [126]. Elevated ROS can activate redox-sensitive stress and inflammatory signaling pathways, including the nuclear factor-κB (NF-κB) and MAPK pathways [127,128]. Activation of these pathways enhances inflammatory cytokine production, cellular injury, and profibrotic signaling [129,130], thereby reinforcing the interaction among hypoxia, inflammation, and tissue remodeling.

Aging is associated with progressive mitochondrial dysfunction and increased oxidative stress, accompanied by shifts in macrophage phenotypes and inflammatory signaling, dysregulation of the complement system, and impaired tissue repair. Collectively, these age-related changes create a tissue microenvironment that promotes pathological angiogenesis and fibrosis. Oxidative stress is a major risk factor for AMD, together with metabolic dysfunction, complement activation, lipid dysregulation, and chronic inflammation [90].

With aging and cumulative exposure to oxidative stress, RPE function progressively deteriorates [131,132]. This deterioration impairs the clearance of metabolic byproducts [133,134]. It disrupts the integrity of Bruch’s membrane, contributing to the accumulation of lipid- and protein-rich extracellular deposits, known as drusen, between the RPE and Bruch’s membrane [90]. The RPE is particularly susceptible to metabolic and oxidative stress because it relies heavily on mitochondrial oxidative metabolism and fatty acid β-oxidation to meet its own energy demands and support the metabolic requirements of the adjacent neural retina [135,136,137].

Age-associated changes in macrophage metabolism may further exacerbate oxidative stress and chronic inflammation in the outer retina. A metabolic shift toward glycolysis is generally associated with a pro-inflammatory macrophage phenotype and may alter local lipid handling, cytokine production, and inflammatory tone within the subretinal space [138,139]. The resulting interplay among dysfunctional RPE cells, oxidized lipids, and activated macrophages may sustain an inflammatory microenvironment that promotes MNV and subsequent fibrotic remodeling.

Inflammasome signaling represents an important mechanism linking intracellular stress to innate immune activation in AMD. The NLR family pyrin domain-containing 3 (NLRP3) inflammasome can be activated by stress signals associated with AMD, including lysosomal membrane permeabilization, mitochondrial dysfunction, and the accumulation of oxidized lipids [140]. Destabilization of RPE lysosomes can activate the NLRP3 inflammasome, resulting in caspase-1-dependent maturation and release of the IL-1 family cytokines IL-1β and IL-18 [141]. NLRP3 inflammasome activation in RPE cells and infiltrating or tissue-resident myeloid cells may therefore amplify local inflammatory responses.

These inflammatory pathways connect upstream metabolic and oxidative stress to NF-κB-dependent cytokine production, VEGF signaling, and TGF-β-mediated ECM remodeling. Persistent activation of these pathways may promote CNV, macrophage recruitment, fibroblast and myofibroblast activation, and ultimately subretinal fibrosis in nAMD [129,140]. Oxidative stress and cellular senescence may also induce EndMT, providing an additional mechanism through which vascular endothelial cells acquire mesenchymal and fibrogenic properties and contribute to subretinal fibrosis [142].

Redox metabolism also influences pathological retinal angiogenesis. Nicotinamide adenine dinucleotide (NAD^+^) suppresses mitochondria-derived hydrogen peroxide during the transition of proliferating endothelial cells to quiescence. Inhibiting NAD^+^ synthesis has been reported to limit excessive angiogenesis in experimental retinopathy, highlighting the importance of metabolic redox regulation in pathological retinal neovascularization [143].

Advanced glycation end products (AGEs) provide an additional link between metabolic dysfunction, oxidative stress, and fibrosis. AGEs accumulate through nonenzymatic glycation of proteins and lipids, particularly under conditions of chronic hyperglycemia and aging [144,145,146,147]. Binding of AGEs to the receptor for AGE (RAGE) promotes NADPH oxidase-dependent ROS production and activates downstream signaling pathways, including NF-κB, MAPK, the NLRP3 inflammasome, and TGF-β signaling [148,149,150]. In DR, persistent AGE–RAGE signaling contributes to endothelial dysfunction, inflammation, BRB disruption, and ECM remodeling, thereby creating a microenvironment that supports pathological angiogenesis and FVM formation [145,146].

In nAMD, the age-related accumulation of lipids, AGEs, and extracellular deposits within the RPE-Bruch’s membrane complex may contribute to RPE dysfunction, including mitochondrial impairment, defective lysosomal degradation, and a reduced capacity to clear metabolic waste [151,152,153]. These alterations promote the accumulation of extracellular deposits, including drusen, and sustain chronic cellular stress. AGEs may also directly cross-link ECM proteins, altering matrix organization, increasing tissue stiffness, and reducing susceptibility to proteolytic degradation [154,155]. These AGE-induced changes in matrix mechanics and turnover may further promote a tissue microenvironment that is permissive to fibrotic remodeling [155].

#### 4.3.3. Complement Activation

Dysregulation of the complement system represents another hallmark of AMD pathogenesis. Complement components are abundant in drusen, and persistent complement activation contributes to chronic inflammation, retinal injury, and disease progression [156].

**Figure 4:**
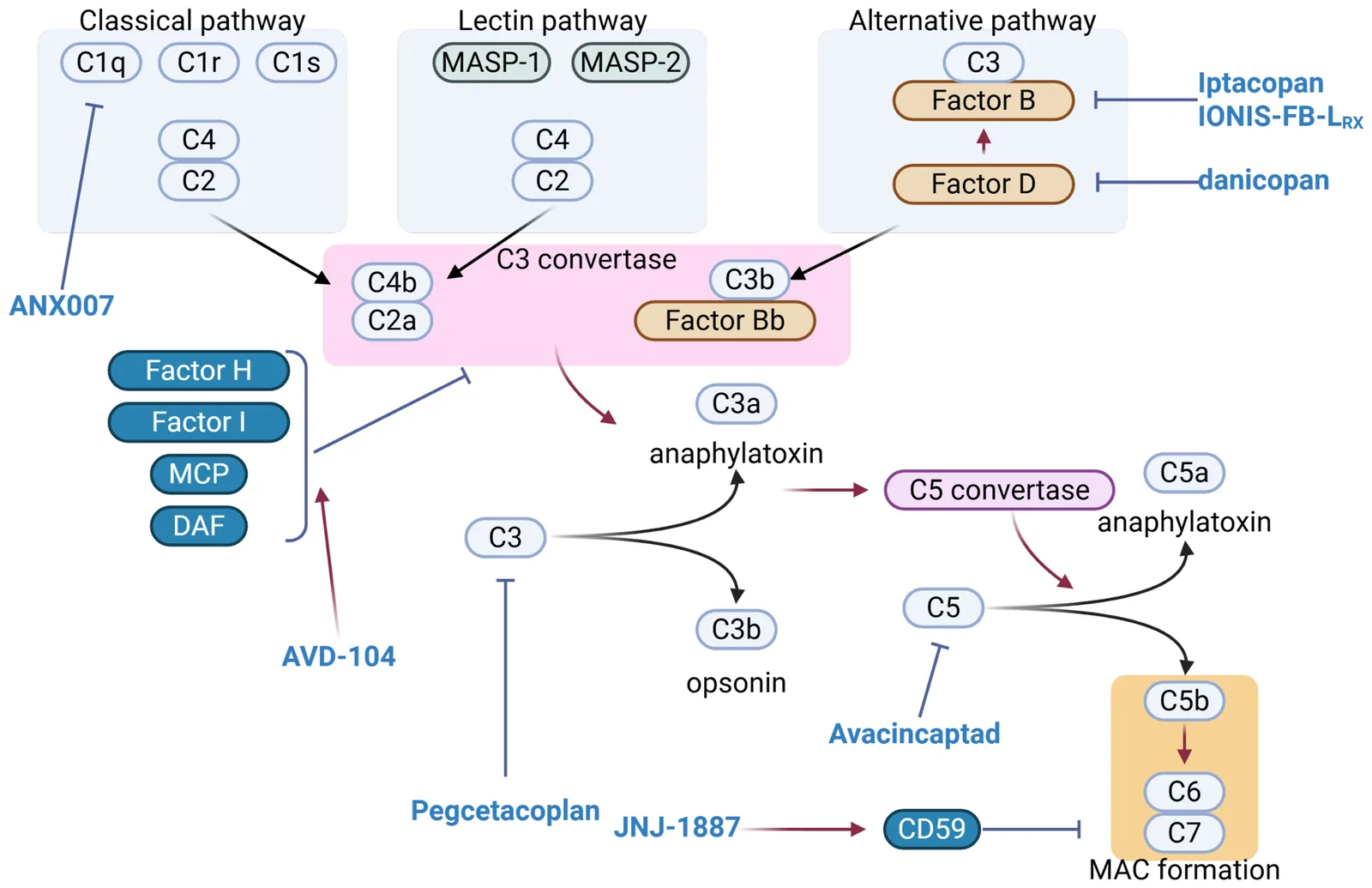
Complement-targeted therapies with potential relevance to fibrotic remodeling in ocular diseases. The classical, lectin, and alternative complement pathways converge on C3 and C5 activation, generating the anaphylatoxins C3a and C5a and ultimately inducing the formation of the membrane attack complex (MAC). These complement effectors may promote inflammatory cell recruitment and activation, thereby contributing to tissue injury and fibrotic remodeling. Therapeutic agents targeting distinct components of the complement cascade include ANX007, iptacopan, IONIS-FB-LRx, danicopan (ACH-4471), pegcetacoplan, and avacincaptad pegol. AVD-104 is proposed to modulate complement regulation via factor H, whereas JNJ-1887 promotes soluble CD59 expression to inhibit the formation of the membrane attack complex. Although these agents have been evaluated primarily for GA, their potential effects on ocular fibrosis remain largely unexplored. Blue bars indicate inhibition, and red arrows indicate activation or enhancement of complement-regulatory activity. Created in BioRender. Yamada, K. (2026) https://BioRender.com/ns9btcu.

The complement system is activated through three pathways: the classical, lectin, and alternative pathways (Figure 4). Although different mechanisms initiate these pathways, they converge on the cleavage of C3. The classical and lectin pathways generate the C3 convertase C4b2a, whereas the alternative pathway generates C3bBb. These C3 convertases cleave C3 into C3a and C3b. C3a acts as an anaphylatoxin that promotes inflammation, whereas C3b functions as an opsonin that facilitates the recognition and clearance of target molecules and damaged cells. C3b also contributes to the formation of C5 convertases, which cleave C5 and initiate the terminal complement pathway (Figure 4) [156]. C5a is a potent anaphylatoxin and chemoattractant, whereas C5b subsequently associates with C6, C7, C8, and C9 to form the membrane attack complex (MAC) [156]. Depending on its abundance and cellular context, MAC deposition can induce cell activation, membrane injury, or cell lysis.

In healthy eyes, complement activation is tightly controlled by soluble and membrane-bound endogenous regulators, including complement factor H (FH), factor I (FI), membrane cofactor protein (MCP/CD46), decay-accelerating factor (DAF/CD55), and CD59. FH is a major regulator of the alternative pathway. It limits the formation and stability of the alternative pathway C3 convertase, C3bBb, and acts as a cofactor for FI-mediated cleavage of C4b. MCP also acts as a cofactor for FI, promoting the proteolytic inactivation of both C3b and C4b and thereby regulating the alternative and classical/lectin pathways, respectively. DAF accelerates the dissociation of both the alternative pathway and classical/lectin pathway C3 and C5 convertases. In contrast, CD59 acts at the terminal stage of the cascade by inhibiting C9 incorporation and MAC assembly [156,157,158,159].

These complement regulators exhibit distinct distributions within the eye. DAF has been detected in the retinal nerve fiber layer, whereas MCP is localized predominantly to the basolateral surface of the RPE [160,161]. CD59 is expressed in the cornea, retina, and choroid, where it protects ocular cells from terminal complement-mediated injury [162]. FH is found predominantly in the choriocapillaris, whereas FI has been detected primarily in the inner retina [163]. In Bruch’s membrane, factor H-like protein 1 (FHL-1), an alternative spliced product of the *CFH* gene, appears to be the predominant complement regulator because it can diffuse through Bruch’s membrane more readily than full-length FH [164].

Genetic evidence strongly supports a role for complement dysregulation in AMD. Several AMD-associated variants have been identified in genes encoding complement components and regulatory proteins. The common Y402H polymorphism in *CFH*, which encodes both FH and FHL-1, is located within a glycosaminoglycan (GAG)-binding region. The risk-associated 402H variant reduces FHL-1 heparan sulfate binding in Bruch’s membrane [164], thereby impairing local regulation of the alternative pathway and increasing susceptibility to complement-mediated inflammation [165]. AMD-associated variants in C3 may similarly promote complement activation by increasing C3 activity, reducing its interaction with FH, or decreasing FH- and FI-mediated C3b inactivation [166,167,168,169].

Complement activation may contribute to pathological angiogenesis and fibrosis through several downstream mechanisms. Complement-derived effectors, particularly C3a and C5a, activate and recruit macrophages and microglia and stimulate the production of inflammatory cytokines, VEGF, and TGF-β. These responses can promote endothelial activation, inflammatory cell accumulation, and ECM remodeling, thereby creating a microenvironment that favors CNV and subretinal fibrosis in nAMD [170,171]. Activated microglia may further amplify complement signaling by producing complement components, thereby establishing a positive feedback loop between complement activation and retinal inflammation [172].

Complement dysregulation has also been implicated in DR. Vitreous samples from patients with PDR show increased concentrations of C3 and activated C3 fragments, including C3b, consistent with enhanced intraocular complement activation [173]. Increased vitreous C3a/C3, C5a/C5, and Ba/factor B ratio further support local activation of C3, C5, and the alternative pathway in PDR. Diabetic retinal tissues also exhibit reduced expression of the complement regulators DAF and CD59 in retinal vessel walls [174] and increased MAC deposition in retinal vessels [174] and the choriocapillaris [175]. Loss of complement regulation may therefore increase endothelial injury, leukocyte recruitment, and BRB dysfunction. Although these changes are consistent with a microenvironment permissive for pathological angiogenesis and fibrovascular remodeling, the direct mechanisms linking complement activation to fibrosis in PDR remain incompletely defined.

Complement activation also increases during retinal aging and oxidative injury. Microglia and recruited monocytes in aged retinas show increased local expression of C3, and the abundance of C3-expressing myeloid cells correlates with outer nuclear layer thinning and inflammatory chemokine expression [176]. Similarly, experimental models of photo-oxidative retinal injury demonstrate activation and migration of resident microglia and macrophages toward the outer retina, accompanied by deposition of C3, factor B, and MAC in the outer retina and RPE [177,178]. These findings support a reciprocal relationship in which oxidative stress activates complement signaling, while complement-derived inflammatory mediators further amplify retinal inflammation and tissue injury.

#### 4.3.4. Inflammatory Cytokine and Chemokine Signaling

Oxidative stress, AGE–RAGE signaling, and complement activation can each promote the production of inflammatory, angiogenic, and profibrotic mediators [146,156,179]. Pro-inflammatory cytokines, such as IL-1β, IL-6, and TNF-α, together with angiogenic and profibrotic mediators, promote crosstalk among endothelial cells, RPE cells, macrophages, microglia, and mesenchymal cells. These interactions establish a chronic inflammatory microenvironment within the retina and choroid, thereby linking persistent inflammation to pathological angiogenesis and fibrotic remodeling in nAMD and PDR [5,61,75].

Chemokines act as chemoattractants, recruiting circulating immune cells to sites of retinal and choroidal injury. Recent studies suggest that infiltrating monocytes, rather than resident microglia, are major contributors to CNV and fibrotic remodeling [63,180,181,182,183,184]. Consistent with this concept, microglial depletion has shown minimal effects on lesion development [184], whereas depletion of choroidal resident macrophages exacerbates fibrosis [185]. Collectively, these findings support a model in which monocyte-derived macrophages promote chronic inflammation and fibrosis, while at least some populations of resident macrophages exert homeostatic or protective functions.

Circulating monocytes express C-C motif chemokine receptor 2 (CCR2), which binds C-C motif chemokine ligand 2 (CCL2), also known as monocyte chemoattractant protein-1 (MCP-1). The CCL2–CCR2 signaling axis promotes monocyte recruitment, after which infiltrating CCR2^+^ monocytes differentiate into macrophages within retinal and subretinal tissues [186]. In an experimental model of subretinal fibrosis, depletion of CCR2+ monocytes from days 5 to 10 after the second laser treatment significantly reduced both the vascular and fibrotic components of the lesion [184], supporting a direct contribution of recruited monocytes to pathological angiogenesis and fibrotic remodeling.

CCL2 signaling has also been implicated in DR and DME, together with other chemokines, including CCL8, C-X-C motif chemokine ligand 8 (CXCL8), and CXCL10. Among these chemokines, CCL2 and CXCL10 have been identified as potentially important contributors to this pathogenesis [187]. In addition to recruiting monocytes, CCL2 can act directly on vascular endothelial cells by activating PKC-dependent signaling pathways and disrupting the organization of tight junction proteins, such as occludin and ZO-1 [188]. Thus, CCL2–CCR2 signaling may contribute to retinal and choroidal disease through both the recruitment of inflammatory monocytes and the direct impairment of endothelial barrier integrity.

Resident microglia express C-X3-C motif chemokine receptor 1 (CX3CR1). Its ligand, C-X3-C motif chemokine ligand 1 (CX3CL1), also known as fractalkine, exists in two forms: a membrane-bound form that functions as an adhesion molecule and a soluble form that acts as a chemoattractant [189]. CX3CL1–CX3CR1 signaling regulates microglial migration, activation, and intracellular signaling responses [190,191]. In diabetic mice, the accumulation of CD11b^+^ myeloid cells in retinal capillaries is increased and is accompanied by elevated CCR5 expression in circulating CD11b^+^ cells and increased CX3CR1 expression in resident retinal microglia [192].

The CX3CL1–CX3CR1 axis appears to have context-dependent roles in DR, influencing retinal inflammation and angiogenesis while also supporting tissue homeostasis and neuroprotection [193]. Although elevated levels of soluble CX3CL1 have been associated with retinal inflammation and endothelial dysfunction, disruption of CX3CL1–CX3CR1 signaling can aggravate retinal injury. CX3CL1 deficiency exacerbates the severity of experimental DR, whereas exogenous CX3CL1 administration reduces inflammation, oxidative stress, and neuronal damage [194]. Consistent with these findings, CX3CR1 deficiency in diabetic mouse retinas is associated with enhanced microglial activation, neuronal loss, astrogliosis, and increased extravasation of fibrin and fibrinogen into the retinal tissue [191,194,195]. Collectively, these observations suggest that intact CX3CL1–CX3CR1 signaling restrains excessive microglial activation and preserves neurovascular homeostasis under diabetic conditions. However, dysregulated CX3CL1 signaling may contribute to inflammation and endothelial dysfunction depending on its local concentration, molecular form, and disease stage.

In addition to CCL2–CCR2 and CX3CL1–CX3CR1 axes, multiple pro-inflammatory cytokines and chemokines contribute to neovascular inflammation and barrier dysfunction. Stimulation with TNF-α, IL-1β, and IFN-γ induces an inflammatory phenotype in brain pericytes through activation of the NF-κB and STAT1 signaling pathways, whereas PDGF-BB suppresses this inflammatory response [196]. TNF-α can also promote fibrotic remodeling by inducing EMT in RPE cells [197,198,199].

CXCL8, also known as IL-8, signals through the chemokine receptors CXCR1 and CXCR2, and promotes neutrophil recruitment to the retinal vasculature [200]. In response to inflammatory cytokines and chemokines, retinal endothelial cells upregulate surface adhesion molecules, including ICAM-1 and VCAM-1, thereby facilitating leukocyte adhesion to retinal capillaries and subsequent extravasation [201]. CXCL8 can further compromise vascular barrier integrity by inducing the internalization of VE-cadherin from the endothelial cell junction [202].

In particular, MCP-1/CCL2-mediated recruitment of circulating monocytes may promote the accumulation of profibrotic macrophage populations, including SPP1-expressing cells, within neovascular lesions. Bulk RNA-seq of mouse macrophages treated with succinate or lactate in acidic hypoxia identified *Spp1* as a key fibrosis-associated gene. SPP1+ macrophages expand in early to mid CNV and in SRF. SPP1 binds to CD44, thereby activating the RhoA/YAP1 pathway, facilitating pro-fibrotic gene transcription, and promoting EMT-like changes in RPE [203].

Collectively, these cytokine- and chemokine-mediated responses promote inflammatory activation of neurovascular cells, leukocyte recruitment, disruption of the endothelial barrier, and fibrotic transformation, thereby reinforcing the chronic inflammatory microenvironment that drives retinal vascular disease.

### 4.4. Mechanical Signaling and ECM-Dependent Mechanotransduction

#### 4.4.1. ECM Composition and Stiffness

The fibrin-rich ECM is progressively replaced by cellular fibronectin and type III and type I collagens produced by initially non-contractile matrix fibroblasts [204]. During scar formation in nAMD, collagen microfibrils self-assemble into higher-order structures, forming fibrils and subsequently fibers that are stabilized by covalent cross-linking [204,205]. Subpopulations of collagen-producing fibroblasts exhibit high expression of molecules involved in ECM synthesis and degradation, as well as collagen fiber assembly, thereby promoting fibrotic remodeling [205].

As the collagen-rich ECM becomes denser and stiffer, it promotes the assembly of contractile actomyosin bundles, or stress fibers, in fibroblasts and facilitates their differentiation into myofibroblasts [204,206]. The increased contractile forces generated by these myofibroblasts further stiffen the scar ECM, enabling it to withstand external mechanical forces and restore the affected tissues’ mechanical integrity. ECM stiffness is further increased by fibroblast- and inflammatory cell-mediated chemical cross-linking [204].

In turn, increased ECM stiffness activates mechanotransduction pathways that regulate a broad range of cellular functions, including cell adhesion, migration, survival, proliferation, and differentiation [207]. Thus, reciprocal interactions between ECM stiffening and fibroblast contractility may establish a self-reinforcing mechanical feedback loop that promotes fibrosis progression and persistence.

#### 4.4.2. Integrin-FAK/SRC Signaling

Cells sense substrate stiffness through integrin-based adhesion complexes that mechanically link the ECM to the actin cytoskeleton [208]. Integrin β1 heterodimerizes with several α-integrin subunits, forming collagen receptors that regulate fibroblast function during tissue repair [209]. Among these receptors, genetic deletion of integrin α11β1, but not of the other collagen-binding integrins α1β1 or α2β1, attenuated fibroblast activation and reduced dermal scarring [210].

Several integrins recognize the arginine–glycine–aspartate (RGD) motif present in multiple ECM proteins, including vitronectin and fibronectin. RGD-binding integrins, such as α5β1, αvβ3, and αvβ5, mediate fibroblast mechanosensing, force transmission, and myofibroblast activation [211,212,213]. In addition, αv-containing integrins, including αvβ1, αvβ3, αvβ5, and αvβ6, can mechanically activate latent TGF-β1 by transmitting cytoskeletal pulling forces to the RGD motif within the latency-associated peptide (LAP) [214,215]. These integrins contribute to fibrotic remodeling, as pharmacological inhibition or genetic deletion of specific αv integrins suppresses fibrosis in several experimental animal models [215,216,217].

Basement membranes can also promote the rapid and robust assembly of fibronectin fibrils through sliding focal adhesions driven by a contractile winch mechanism [218]. Within focal adhesions, integrin engagement and mechanical loading activate focal adhesion kinase (FAK) and SRC family kinases, which transmit mechanical signals to downstream pathways that regulate cytoskeletal organization, cell migration, survival, proliferation, and fibroblast differentiation. Consistent with this role, loss of FAK function in cultured fibroblasts attenuates myofibroblast activation [219]. Thus, integrin-FAK/SRC signaling provides an important molecular link between ECM stiffness, actomyosin contractility, and sustained fibroblast activation during fibrotic remodeling.

#### 4.4.3. RhoA/ROCK Signaling and Actomyosin Contractility

Myofibroblast contraction is driven by interactions between actin filaments and nonmuscle myosin II and is regulated by phosphorylation of the myosin light chain (MLC) [220,221]. Myosin light chain kinase (MLCK) phosphorylates MLC, thereby promoting myosin II activation, actomyosin bundle assembly, and force generation. Conversely, MLC phosphatase (MLCP) dephosphorylates MLC, reducing actomyosin interactions and contractile activity [222].

The Rho/Rho-associated coiled-coil-containing protein kinase (ROCK) is a major regulator of actomyosin contractility [204]. The GTP-bound RhoA activates ROCK and enhances MLC phosphorylation through two complementary mechanisms. ROCK can directly phosphorylate MLC and inhibit MLCP by phosphorylating its regulatory subunit, thereby sustaining myosin II activity and actomyosin-generated tension [220,221]. Increased actomyosin contractility, in turn, reinforces focal adhesions and facilitates the transmission of intracellular forces to the surrounding ECM. Thus, RhoA/ROCK signaling connects mechanical cues from the ECM to cytoskeletal tension and the acquisition and maintenance of the contractile myofibroblast phenotype.

#### 4.4.4. Hippo-YAP/TAZ Signaling

TGF-β, Wnt, and YAP/TAZ signaling pathways converge on shared transcriptional programs that integrate multiple fibrotic signals and promote fibroblast activation [70]. YAP and TAZ are transcriptional coactivators regulated by the Hippo pathway. They can respond to various upstream biochemical and mechanical stimuli relevant to myofibroblast activation and fibrosis, including Wnt signaling and G protein-coupled receptor (GPCR) ligands [204].

The core Hippo kinase cascade comprises the mammalian STE20-like kinases 1 and 2 (MST1 and MST2; also known as serine/threonine protein kinases 4 and 3, respectively), large tumor suppressor kinases 1 and 2 (LATS1 and LATS2). When activated, MST1/2 promote the activation of LATS1/2, which subsequently phosphorylate YAP and TAZ. Phosphorylated YAP and TAZ are retained in the cytoplasm and may undergo ubiquitin-mediated proteasomal degradation, thereby limiting their transcriptional activity [223].

Mechanical cues generated during ECM remodeling can regulate YAP/TAZ activity through integrin–FAK signaling, contributing to the persistent activation of fibroblasts and myofibroblasts [224,225]. YAP/TAZ activity is sensitive to mechanical stimuli, including fluid shear stress in endothelial cells [226] and changes in matrix stiffness [227]. Both YAP and TAZ preferentially localize to the nucleus in fibroblasts cultured on stiff substrates that mimic fibrotic tissue or exposed to mechanical stress. In contrast, they remain predominantly cytoplasmic in fibroblasts cultured on physiologically soft substrates [227].

Increased matrix stiffness promotes integrin engagement, focal adhesion assembly, and RhoA-dependent actomyosin contractility, thereby facilitating YAP/TAZ activation and nuclear accumulation. RhoA activation and the resulting formation of actomyosin stress fibers can reduce LATS1/2-mediated inhibition of YAP/TAZ and increase the cytoplasmic pool of unphosphorylated YAP and TAZ available for nuclear translocation. In addition, cytoskeletal tension can facilitate YAP nuclear entry through mechanically induced changes in nuclear architecture and nuclear pore transport [228]. Once in the nucleus, unphosphorylated YAP and TAZ interact with members of the TEA domain family of DNA-binding transcription factors (TEAD) and induce the expression of genes associated with fibroblast activation and fibrotic remodeling [204]. These target genes include CCN2, also known as CTGF, which promotes fibroblast activation, ECM production, and tissue fibrosis.

Collectively, these mechanisms identify YAP/TAZ as important mechanosensitive transcriptional regulators that connect ECM stiffness and cytoskeletal tension to sustained fibroblast activation. Crosstalk between YAP/TAZ, TGF-β, and Wnt signaling further reinforces profibrotic transcriptional programs, promoting myofibroblast activation and ECM deposition [70]. This feed-forward relationship may contribute to the persistence and progression of fibrosis in nAMD and PDR.

## 5. Clinical Approaches and Therapeutic Strategies

### 5.1. Current Clinical Management

Effective clinical management of nAMD and DR starts with early detection and continuous structural monitoring. As described in Section 2, persistent or treatment-resistant SHRM is an important imaging biomarker associated with fibrotic progression and poor visual outcomes in nAMD [20,21,22]. Hyperreflective foci and fibrovascular pigment epithelial detachment may provide additional evidence of inflammatory and fibrovascular remodeling, although these findings are not specific to fibrosis [229]. High-resolution OCT is indispensable for detecting and monitoring retinal and subretinal fluid, pigment epithelial detachment, retinal thickening, and fibrovascular changes. OCT angiography (OCTA) provides complementary, noninvasive visualization of retinal and choroidal microvasculature and can identify non-exudative MNV before the development of fluid or hemorrhage. In DR, OCT and OCTA facilitate the assessment of DME, retinal ischemia, and microvascular abnormalities. Together, these imaging modalities help guide the timing and frequency of interventions and may identify neovascular or fibrovascular changes before irreversible scarring occurs.

Once active neovascularization or exudation is detected, intravitreal anti-VEGF therapy remains the gold standard for nAMD, DME, and selected stages of DR. Pharmacological treatments, such as ranibizumab, bevacizumab, and aflibercept, have substantially improved clinical outcomes by suppressing pathological angiogenesis and reducing both subretinal and intraretinal fluid [230]. Ranibizumab (Lucentis, Genentech, South San Francisco, CA, USA), a humanized monoclonal antibody Fab that binds VEGF-A, was approved for nAMD in 2006 [15,231]. Aflibercept (Eylea, Regeneron Pharmaceuticals, Tarrytown, NY, USA), also known as VEGF Trap-Eye, is a recombinant fusion protein containing extracellular domains of VEGFR1 and VEGFR2 that binds VEGF-A, VEGF-B, and placental growth factor. It was approved for nAMD in 2011 [16]. Bevacizumab (Avastin, Genentech), a full-length anti-VEGF-A antibody, is widely used off-label for nAMD, PDR, and DME. More recent options include brolucizumab (Beovu, Novartis, Basel, Switzerland), approved in 2019 [232], and the ranibizumab biosimilar ranibizumab-runa (byooviz, Biogen, Cambridge, MA, USA, and Samsung Bioepis, Incheon, Republic of Korea) [233,234], approved in 2021. Faricimab and high-dose aflibercept have further expanded the available options for extending treatment intervals in nAMD and DME. Faricimab was approved in 2022 [235,236], while aflibercept 8 mg can be administered at intervals of up to 16 weeks in appropriately responsive patients [237].

However, the chronic nature of these diseases necessitates frequent intravitreal injections, which impose a substantial burden on patients, caregivers, and healthcare systems. Clinical practice has therefore shifted towards individualized dosing strategies, such as the treat-and-extend (T&E) protocols. Under this approach, the interval between injections is gradually extended when disease activity remains controlled and shortened when fluid, hemorrhage, or other signs of reactivation recur. Nevertheless, some patients develop fibrovascular scarring or geographic atrophy (GA) despite effective control of exudation, contributing to long-term vision loss [238].

Long-acting delivery systems offer another strategy for reducing treatment burden. The ranibizumab Port Delivery System (PDS) (Susvimo, Genentech) was approved by the US Food and Drug Administration (FDA) in 2021 and provides continuous intraocular drug delivery through a surgically implanted, refillable reservoir [239]. Following a voluntary recall related to implant performance, an updated device was reintroduced in the United States in 2024 and is generally refilled every 6 months. Susvimo was subsequently approved for DME in February 2025.

Clinical management is further complicated by substantial interindividual variability in the response to anti-VEGF therapy. Genetic variants and systemic, ocular, and lesion-specific characteristics have been associated with persistent subretinal fluid, fibrovascular pigmented epithelium detachment, and reduced treatment responsiveness [240]. However, pharmacogenetic testing is not currently part of routine treatment selection. In patients with persistent disease activity despite adequate treatment, clinicians may increase treatment frequency, reassess the diagnosis, or switch to another anti-VEGF agent. Switching to an alternative anti-VEGF agent is a commonly used clinical option for patients with an inadequate response to the initial treatment, although the benefits may vary depending on the agent and are often more evident in anatomical than visual outcomes [241,242,243].

To address the limitations of single-target therapies and extend treatment durability, the clinical trend is shifting toward bispecific antibodies. Faricimab (Vabysmo, Genentech) is a bispecific antibody that inhibits both VEGF-A and ANG2. By targeting VEGF-mediated permeability and ANG2-mediated vascular instability, faricimab is intended to promote vascular stability while maintaining antiangiogenic activity. This dual-inhibition mechanism allows for safely extended dosing intervals of up to 16 weeks in many patients without compromising visual or anatomical outcomes compared to standard therapies [244]. Faricimab is therefore an additional option for treatment-naïve patients and for patients experiencing chronic exudation or a high injection burden during conventional anti-VEGF treatment. However, evidence from switching studies is derived largely from observational cohorts, and not all previously treated patients achieve longer intervals or additional visual improvement [241]. The FDA-approved indication includes both nAMD and DME.

In addition to targeting angiogenesis, clinical management often requires addressing the inflammatory cascades that influence disease progression. Chronic inflammation is a significant driver in both nAMD and DR, worsening vascular permeability. In cases of DME, clinicians utilize intravitreal corticosteroids, such as sustained-release dexamethasone implants. These treatments provide an alternative for selected patients, including those with an inadequate response to anti-VEGF therapy, pseudophakic eyes, or difficulty maintaining frequent injection schedules. Corticosteroids suppress inflammatory cytokine production and can reduce retinal thickness and edema. Dexamethasone implants may provide substantial short-term anatomical improvement in anti-VEGF refractory DME, including selected eyes with an associated epiretinal membrane (ERM) [245]. Their use must be balanced against the risks of elevated intraocular pressure and cataract formation.

Laser photocoagulation and panretinal photocoagulation also remain relevant in diabetic eye disease [246]. Focal or grid laser treatment now has a more limited role in center-involving DME but may be considered for selected non-center-involving lesions [247]. Panretinal photocoagulation remains an established treatment for PDR, particularly when frequent follow-up or repeated anti-VEGF treatment may not be feasible. Anti-VEGF therapy can also induce regression of retinal neovascularization, but sustained monitoring is necessary because neovascularization can recur when treatment is interrupted [246,248].

When pharmacological and laser treatments fail to control the disease, or for patients who present in advanced stages, surgical management becomes necessary. In severe PDR, fibrovascular proliferation frequently leads to vitreoretinal traction, vitreous hemorrhage, or tractional retinal detachment (TRD). Pars plana vitrectomy (PPV) is performed to carefully dissect and sever the fibrotic bands, relieving traction and reattaching the retina [249]. ERM peeling may also be performed when clinically significant tangential traction contributes to macular distortion or edema [245].

Surgery has a substantially more limited role in nAMD. Submacular surgery is not routinely performed to remove established subfoveal fibrovascular lesions because clinical trials have not demonstrated a consistent visual benefit compared with observation, and the procedure carries a risk of additional retinal and retinal pigment epithelial damage [250]. Selected surgical procedures may be considered for uncommon complications, particularly large submacular hemorrhage, but the optimal approach remains uncertain [251]. Established subretinal fibrosis is therefore managed primarily through prevention of recurrent exudation, visual rehabilitation, and low-vision support rather than surgical excision [23].

In addition to the clinical management of nAMD, PDR, and DME, complement-targeted therapies have entered clinical practice for GA secondary to AMD. Complement activation may contribute to fibrotic remodeling; however, the effects of these therapies on ocular fibrosis have not been investigated. Avacincaptad pegol (Izervay, Iveric Bio, Parsippany, NJ, USA) is a pegylated RNA aptamer that inhibits complement C5 and is approved for the treatment of GA secondary to AMD (Figure 4). In the phase 3 GATHER2 trial (NCT04435366), monthly intravitreal administration reduced GA lesion growth by 14% compared with sham at 12 months, with a sustained treatment effect through 2 years [252,253]. However, the anatomical benefit has not translated into clear improvement in visual function, and treatment is associated with an increased risk of nAMD.

Pegcetacoplan, also known as APL-2 (Syfovre; Apellis Pharmaceuticals, Waltham, MA, USA), is a PEGylated compstatin-derived peptide inhibitor of complement C3. Its systemic formulation is marketed as Empaveli for paroxysmal nocturnal hemoglobinuria [254], whereas its intravitreal formulation is marketed as Syfovre for GA secondary to AMD. Pegcetacoplan binds with high affinity to C3 and C3b, thereby inhibiting C3 cleavage and the generation of downstream complement effectors. In phase 3 OAKS and DERBY trials for GA (NCT03525613, NCT03525600), intravitreal Pegcetacoplan reduced the rate of lesion growth compared with sham treatment. However, it did not significantly improve key visual function outcomes at 24 months [255]. Both avacincaptad and Pegcetacoplan are approved for GA, although their potential effects on fibrotic remodeling have not been investigated.

Overall, current clinical management effectively suppresses neovascular activity, reduces edema, and preserves vision in many patients. However, existing treatments do not reliably prevent or reverse established fibrovascular scarring [23,256]. Early diagnosis, sustained control of exudation and inflammation, individualized treatment intervals, and timely management of tractional complications, therefore, remain central to limiting irreversible structural damage.

### 5.2. Current Limitations

Despite the success of anti-VEGF therapies in reducing macular exudation, these treatments often fail to prevent the ultimate progression to subretinal fibrosis. This phenomenon is caused by the paradoxical effect of anti-VEGF treatment, in which the continuous suppression of angiogenesis inadvertently promotes a profibrotic microenvironment. In many cases, patients successfully treated for exudative fluid still experience fibrovascular scarring, with nearly 40% developing significant subretinal fibrosis after a decade of ongoing treatment. Imaging studies utilizing OCTA have further demonstrated that while anti-VEGF therapy successfully regresses small vessels, the neovascular network remodels, leading to a significant increase in the density of subretinal fibrotic lesions. This neovascular remodeling and fibrosis serve as structural biomarkers predicting an incomplete response to anti-VEGF treatment in nAMD patients, indicating that anti-VEGF agents fail to prevent the maturation of the fibrotic scaffold [257].

The transition from active neovascularization to subretinal fibrosis creates devastating structural consequences for the retina. The origin of this scarring involves the transdifferentiation of both the RPE and migrating macrophages into ECM-producing myofibroblasts [258]. As these myofibroblasts deposit excessive layers of collagen and fibronectin, they disturb the microenvironment of the fundus and cause damage to the choroidal and retinal microstructures. This leads to the formation of fibrotic scars and the death of photoreceptor cells, resulting in permanent and irreversible vision loss regardless of whether the macula is free of exudative fluid.

Beyond these biological limitations, the chronic nature of current therapies imposes a large logistical and financial burden that undermines clinical efficacy. Real-world adherence to anti-VEGF treatment plans is notably lower compared to controlled clinical trials. The Intelligence Research in Sight (IRIS^®^) registry data reveal that nearly 1 in 9 patients with nAMD are eventually lost to follow-up, and 1 in 7 demonstrated non-persistence with their prescribed treatment [259]. This compliance gap is driven largely by treatment fatigue and logistical barriers, such as age, long commuting distances, and the need for an escort to clinical appointments [260]. Consequently, active lesions are frequently undertreated, giving the disease ample time to progress into irreversible stages. Furthermore, clinical burden is worsened by a significant number of patients who exhibit an incomplete response to anti-VEGF therapy. Despite standardized treatments, many patients still suffer from persistent disease activity characterized by ongoing exudation and progressive lesion fibrosis, allowing damage to accrue. In contrast, patients endure chronic, yet ineffective, injection regimens [261].

Finally, the transition of novel anti-fibrotic therapies into clinical practice is fundamentally hindered by the limitations of standard in vivo models. The widely used single-stage laser-induced CNV model is inherently limited because acute lesions naturally regress in wild-type mice, thereby failing to capture the chronic, progressive subretinal fibrosis characteristic of human disease [262]. Furthermore, standard laser-induced lesions primarily elicit an acute wound-healing response following the rupture of Bruch’s membrane. To better recapitulate persistent subretinal fibrosis, a two-stage laser injury model involving repeated laser injury at the same site has been developed and is increasingly used for the evaluation of fibrotic remodeling [263]. In this model, collagen I deposition is increased as early as 3 days after the second laser injury [184]. Whereas collagen-expressing cells gradually disappear after approximately 12 days in the single laser model, repeated injury results in their sustained accumulation [205]. Accordingly, the two-stage model has been used to evaluate potential anti-fibrotic interventions, including MMP12 inhibition [59] and genetic deficiency of granzyme B [264], both of which attenuated subretinal fibrosis. Although this model produces more persistent fibrosis than the single-stage model, it still depends on acute mechanical injury in otherwise healthy animals and does not fully recapitulate the chronic, age-associated pathogenesis of human nAMD. Moreover, evaluations of laser-induced healing have revealed that temporal patterns of key fibrotic events, including EMT and ECM deposition, vary significantly with animal age [265]. These limitations highlight the difficulty of using acute mechanical injury in rodents to faithfully reproduce the chronic, age-driven pathology observed in human nAMD. To properly test emerging anti-fibrotic compounds, researchers are increasingly recognizing the need for time-dependent, late-phase models that distinguish persistent fibrosis from active neovascularization, thereby providing a more accurate representation of the human disease [262].

### 5.3. Potential Anti-Fibrotic Strategies

#### 5.3.1. Drugs in Clinical Trials Targeting Angiogenesis and Vascular Instability

Because fibrosis in nAMD frequently develops following or in association with neovascularization, inhibition of angiogenic pathways may indirectly reduce fibrosis development or progression. Accordingly, several agents targeting angiogenesis have been evaluated in clinical trials (Table 1) [266]. These include approved or late-stage anti-VEGF biosimilars, such as CKD-701/LucenBS (Chong Kun Dang Pharmaceutical, Seoul, Republic of Korea) [267] and HLX04-O (Shanghai Henlius Biotech, Shanghai, China, and Essex Bio-Technology, Hong Kong, China) [268], as well as agents designed to extend treatment durability through long-acting formulations or sustained ocular expression.

Recent approaches to sustained VEGF inhibition include the long-acting anti-VEGF antibody biopolymer conjugate Tarcocimab tedromer [269,283], gene therapies using adeno-associated virus (AAV) vectors such as ABBV-RGX-314 (REGENXBIO, Rockville, MD, USA, and AbbVie, North Chicago, IL, USA) [270], Ixoberogene soroparvovec (ixo-vec; formerly ADVM-022; Adverum Biotechnologies, Redwood City, CA, USA) [271], and LX102 (Innostellar Biotherapeutics, Shanghai, China) [272]. Another approach involves sustained intraocular delivery of axitinib, a small-molecule tyrosine kinase inhibitor (TKIs) [284], through formulations such as AXPAXLI/OTX-TKI (Ocular Therapeutix, Bedford, MA, USA) [277] and CLS-AX (Clearside Biomedical, Alpharetta, GA, USA) [278].

Broader inhibition of the VEGF family has also been investigated. Sozinibercept (Opthea, Melbourne, Victoria, Australia), which neutralizes VEGF-C and VEGF-D [285], did not improve visual outcomes when added to VEGF-A inhibition in phase 3 trials, leading to discontinuation of its nAMD program [285]. Other approaches under clinical evaluation include the VEGF-A/VEGF-C-targeting bispecific protein IBI333 (Innovent Biologics, Suzhou, China) [273] and the AAV-based therapy 4D-150 (4D Molecular Therapeutics, Emeryville, CA, USA), which combines aflibercept expression with RNA interference targeting VEGF-C [274].

Additional strategies have targeted ANG2 and other regulators of vascular stability. The ANG2-targeting antibody nesvacumab (Regeneron Pharmaceuticals) did not provide additional visual benefit when combined with aflibercept and was not advanced to phase 3 [286]. Bifunctional agents targeting VEGF and ANG2 include VIS-101 (Visara, Morrisville, NC, USA, and NovaBridge Biosciences, Rockville, MD, USA) and EXG102-031 (Exegenesis Bio, Philadelphia, PA, USA) [275], whereas gersizangitide (AsclepiX Therapeutics, Bethesda, MD, USA) inhibits VEGFR2 and activates Tie2 through integrin regulation [276]. Other VEGF-independent strategies include collagen-derived anti-angiogenic peptide Lorutengitide (EB-203; EyeBioKorea, Busan, Republic of Korea) [279], and anti-semaphorin 3A antibody BI 764524 (Boehringer Ingelheim, Ingelheim am Rhein, Germany) [280,281]. Although these approaches may improve vascular control or reduce treatment burden, their direct effects on fibrovascular scarring remain unestablished. Other strategies, including anti-endoglin antibody DE-122 (Santen Pharmaceutical, Osaka, Japan, and TRACON Pharmaceuticals, San Diego, CA, USA) [287], and the ROBO4 agonist DS-7080a (Daiichi Sankyo, Tokyo, Japan) [282], did not demonstrate sufficient clinical benefit to support further development.

Other unsuccessful programs have highlighted the limitations of angiogenesis-directed treatment. Abicipar pegol (Molecular Partners, Zürich-Schlieren, Switzerland) demonstrated durable VEGF-A inhibition but was not approved due to a higher incidence of intraocular inflammation, resulting in an unfavorable benefit-to-risk profile [288]. Collectively, these studies illustrate continued efforts to improve vascular control, treatment durability, and pathway coverage. However, most trials were designed to assess visual and anatomical vascular outcomes rather than fibrosis, and it remains unclear whether these approaches prevent or modify fibrovascular scarring.

#### 5.3.2. Clinical Development of Therapies Targeting Fibrovascular and Fibrosis-Related Pathways

Clinical therapies discussed in this section can be divided into two categories (Table 2). The first comprises agents that primarily target angiogenesis, vascular stability, or inflammation and may only indirectly influence fibrotic remodeling. The second comprises agents with experimental evidence of activity against fibrosis-related mechanisms, such as mesenchymal transition, fibroblast or myofibroblast activation, and ECM production. Importantly, no therapy has yet demonstrated clinical efficacy specifically for the prevention or regression of established subretinal fibrosis in nAMD or fibrovascular fibrosis in PDR.

##### Therapies That May Indirectly Influence Fibrosis

Several investigational therapies primarily target angiogenesis, vascular stability, or inflammation and may therefore affect fibrosis only indirectly. These include Tabirafusp tedromer (Kodiak Sciences, Palo Alto, CA, USA), which targets VEGF and IL-6 [289]; RC28-E/eflimrufusp alfa (RemeGen, Yantai, China), which targets VEGF-A and FGF-2 [305,306]; efdamrofusp alfa (Innovent Biologics), which targets VEGF and complement components [307,308,309]. Although these agents may provide broader control of fibrovascular disease than VEGF inhibition alone, their clinical trials have focused primarily on visual acuity and retinal anatomical outcomes, and direct effects on fibrosis have not been established.

PDGF-directed therapies were developed to inhibit pericyte-supported neovascular stabilization and fibrovascular remodeling. However, neither rinucumab combined with aflibercept [310] nor pegpleranib (Ophthotech Corporation, Princeton, NJ, USA) combined with anti-VEGF therapy [310,311] provided additional benefit in later-stage trials. Similarly, EYP-1901/DURAVYU (EyePoint Pharmaceuticals, Watertown, MA, USA) provides sustained inhibition of VEGFR and PDGFR signaling [283,294,295], but its potential effects on fibrosis have not been evaluated clinically. These results illustrate the limitations of inferring antifibrotic efficacy from vascular or anatomical outcomes alone.

##### Therapies with Experimental Activity Against Fibrosis-Related Mechanisms

A smaller group of agents has experimental evidence of activity against fibrosis-related mechanisms. Fenofibrate (Fournier Pharma SA, Dijon, France), a PPARα agonist, is suppresses inflammatory and profibrotic signaling in experimental retinal models and has reduced DR progression in multiple clinical trials. In the FIELD (ISRCTN#64783481) and phase 3 ACCORD-EYE (NCT00542178) studies, fenofibrate reduced the progression of DR or the need for treatment [296,297]. More recently, the randomized, placebo-controlled LENS trial (NCT03439345) demonstrated a reduced risk of progression to referable DR or diabetic maculopathy [298,299]. However, these trials primarily enrolled patients with nonproliferative or early diabetic retinal disease and did not specifically assess established PDR-associated fibrovascular proliferation, fibrotic remodeling, or tractional complications. Therefore, whether the antifibrotic effects observed experimentally translate to PDR-associated fibrosis remains unknown.

Other approaches have targeted inflammation and bioactive lipid signaling involved in angiogenesis, inflammation, and fibrosis. Canakinumab (formerly ACZ885; Novartis) is a fully human monoclonal antibody that selectively neutralizes IL-1β, a proinflammatory cytokine implicated in macrophage activation, angiogenesis, and fibrotic remodeling [300]. A randomized, double-masked phase 1 trial evaluated the safety and tolerability of a single intravenous infusion of canakinumab in 20 patients with subfoveal CNV secondary to nAMD (NCT00503022). However, clinical results were not publicly reported, and development for nAMD did not progress beyond phase 1. Its effect on subretinal fibrosis has not been clinically established.

Sonepcizumab (iSONEP; formerly LT1009, Lpath, San Diego, CA, USA) is an intravitreally administered humanized monoclonal antibody that neutralizes sphingosine-1-phosphate (S1P), a bioactive lipid implicated in angiogenesis, vascular permeability, inflammation, and fibrosis [312]. However, iSONEP failed to meet the primary and key secondary efficacy endpoints when administered alone or in combination with anti-VEGF therapy in the phase 2a NEXUS trial (NCT01414153), and its clinical development for nAMD did not progress further.

Risuteganib (formerly ALG-1001 or Luminate; Allegro Ophthalmics, San Juan Capistrano, CA, USA) is an intravitreally administered synthetic RGD peptide that modulates multiple integrins involved in angiogenesis, inflammation, and cell–extracellular matrix interactions. It has been clinically evaluated primarily in DME and non-exudative AMD, including a randomized phase 2a trial in intermediate non-exudative AMD (NCT03626636) [303,313]. Although integrin-dependent signaling may contribute to fibrovascular remodeling, the effects of risuteganib on subretinal fibrosis in nAMD have not been clinically established.

Brimapitide (XG-102; Xigen, Lausanne, Switzerland) is a cell-penetrating peptide that inhibits c-Jun N-terminal kinase signaling, a stress-activated pathway implicated in inflammation, fibroblast activation, and extracellular matrix remodeling. A single subconjunctival injection of brimapitide was generally well tolerated and was noninferior to a 21-day course of topical dexamethasone for controlling postoperative ocular inflammation in a phase 2 trial [304]. A subsequent phase 3 study evaluated XG-102 after cataract surgery (NCT02235272, NCT02508337). However, XG-102 has not been clinically evaluated for nAMD-associated subretinal fibrosis, and its direct anti-fibrotic efficacy in the retina remains unestablished.

Complement activation contributes to macrophage recruitment and fibrovascular remodeling (Figure 4), although most complement-directed clinical development has focused on GA rather than fibrosis associated with nAMD or DR. ANX007 (Annexon Biosciences, South San Francisco, CA, USA) is an antibody fragment targeting C1q (Figure 4). In the phase 2 ARCHER trial for GA (NCT04656561), ANX007 demonstrated meaningful improvement in BCVA and has proceeded to a phase 3 clinical trial [172].

In contrast, agents targeting C5, such as tesidolumab (Novartis) [314] and eculizumab (Alexion Pharmaceuticals, Boston, MA, USA) [315,316,317], agents targeting factor D, such as lampalizumab (Genentech) [318] and danicopan (Alexion Pharmaceuticals) [319], agents targeting factor B, such as iptacopan (LNP023, Fabhalta, Novartis) and IONIS-FB-LRx (sefaxersen, Roche, Basel, Switzerland) [302], factor H inhibitor GEM103 (Gemini Therapeutics, Wayland, MA, USA) [320], gene therapy expressing soluble CD59 JNJ-1887 (Johnson & Johnson, New Brunswick, NJ, USA) [321], SIGLEC inhibitor AVD-104 (Aviceda Therapeutics, Cambridge, MA, USA) [322], and human immunoconjugate-1 (hI-con1, subsequently designated ICON-1; Iconic Therapeutics, South San Francisco, CA, USA), targeting tissue factor [323,324] have shown mixed or negative results in GA trials. Because these studies did not evaluate retinal or subretinal fibrosis, they do not currently provide clinical evidence for complement-directed antifibrotic therapy.

Collectively, most therapies evaluated clinically to date have targeted angiogenesis, vascular stability, or inflammation and may influence fibrosis only indirectly. Several agents act on pathways with experimental relevance to mesenchymal transition, fibroblast activation, or ECM remodeling, but thus far, none has demonstrated clinical efficacy specifically against established subretinal fibrosis in nAMD or fibrovascular fibrosis in PDR. Future trials should therefore incorporate fibrosis-specific structural and functional endpoints to determine whether modulation of these pathways prevents fibrosis development or promotes regression of established fibrotic lesions.

#### 5.3.3. Emerging Therapeutic Targets and Preclinical Strategies for Fibrosis

The development of effective anti-fibrotic therapies for nAMD and DR requires shifting the therapeutic focus from angiogenesis to the cellular transitions and ECM remodeling that drive scar formation (Table 3).

At the cellular level, preventing mesenchymal transition of RPE cells, endothelial cells, pericytes, and macrophages has emerged as a primary focus. TGF-β is a master regulator of mesenchymal transition. In fact, treatment with a neutralizing antibody targeting TGF-β before subretinal fibrosis induction and continuous daily treatment prevented the development of subretinal fibrosis in an animal model [86]. Although it has not been clinically evaluated for subretinal fibrosis or nAMD, similar TGF-β antibodies, such as Fresolimumab (Sanofi, Paris, France), have been evaluated in Clinical Trials for breast cancer [330,331], sclerosis [332], osteogenesis imperfecta [333], and myelofibrosis [334].

Furthermore, dual inhibition of the profibrotic TGF-β and Wnt signaling cascades by feeding three-month-old *Vldlr*^−/−^ mice with fenofibrate, a PPARα agonist, for 45 days, has demonstrated significant efficacy in vivo, drastically reducing CTGF expression and collagen deposition [325]. Although fenofibrate has reduced DR progression in clinical trials [296,297,298,299], its effects on fibrovascular membrane formation have been evaluated only in preclinical animal models to date [325].

Notch and ERK1/2 pathways are also implicated in fibrosis. RO4929097 (Roche), a selective γ-secretase inhibitor, has been evaluated in clinical trials as an anticancer agent [335,336,337,338] and subsequently examined in preclinical models of ocular fibrosis. Interrupting Notch and ERK1/2 signaling pathways with RO4929097 (intravitreally injected one day after laser induction) effectively suppressed early initiation or progression of fibrotic responses in laser-induced CNV models in young and aged mice [326].

In PDR, longitudinal multi-omics analyses of aqueous humor are revealing potential mediators of fibrovascular contraction. Proteomic and metabolomic profiling before and 7 days after intravitreal aflibercept identified a biphasic change in galactosamine-6 sulfatase (GALNS), with higher levels in PDR than in cataract controls, but lower levels in eyes with tractional retinal detachment, and a further reduction following anti-VEGF treatment [339]. GALNS silencing in cultured fibroblasts increased α-SMA and collagen I expression and enhanced cell migration and contractility, supporting GALNS depletion as a potential contributor to the profibrotic response following anti-VEGF treatment [339]. However, the study did not determine whether restoration of GALNS activity could prevent fibrovascular contraction. Recombinant GALNS replacement with elosulfase alfa (BMN110, Vimizim, BioMarin Pharmaceutical, Novato, CA, USA) is clinically available for mucopolysaccharidosis IVA, demonstrating that GALNS activity can be therapeutically restored [340]. Nevertheless, elosulfase alfa has not been evaluated in ocular fibrotic remodeling, and studies using GALNS supplementation or overexpression in ocular models will be required before GALNS-directed therapy can be considered a viable strategy for preventing anti-VEGF-associated fibrosis.

Pharmacological modulation of ECM homeostasis may provide another potential approach. The multi-component traditional formulation Mudan granules (treatment at 8–14 weeks after induction of diabetes) attenuated retinal dysfunction and retinal thinning in STZ-induced diabetic mice. Proteomic analysis showed that fibrillin-2 (FBN2), which was reduced in diabetic retinas, was restored following treatment, together with reductions in markers associated with TNF-mediated inflammation, neovascularization, and fibrosis [327]. However, whether FBN2 restoration directly mediates these effects remains unclear.

Whereas FBN2 may contribute to the maintenance of ECM organization, excessive proteolytic activity can promote matrix turnover and amplify fibrotic remodeling. Clinical analyses of patients with nAMD receiving long-term anti-VEGF therapy identified an association between elevated levels of active matrix metalloproteinase-8 (aMMP-8) and greater subretinal fibrotic lesion thickness [341]. These findings nominate aMMP-8 as a potential biomarker and therapeutic target. However, the observational nature of the evidence does not establish whether elevated aMMP-8 is a cause or a consequence of advanced fibrosis. Rather than simply promoting matrix degradation, dysregulated MMP activity may alter basement membrane integrity, release matrix-bound signaling molecules, and initiate compensatory matrix deposition [341].

More direct preclinical evidence supports a role for macrophage elastase MMP12 in subretinal fibrosis. MMP12 was highly expressed in macrophages within fibrotic lesions in both a two-stage laser-induced mouse model and human nAMD donor eyes. The selective MMP12 inhibitor MMP408 dose-dependently suppressed TGF-β-induced MMT in vitro. Twice-daily treatment with MMP408 at 3–8 days after the second laser reduced macrophage infiltration and subretinal fibrosis in vivo, providing pharmacological proof of concept for targeting protease-dependent inflammatory and fibrotic remodeling [59].

Granzyme B (*GzmB*), an extracellular serine protease elevated in nAMD lesions, may similarly connect inflammation, ECM remodeling, and RPE phenotypic transition [264]. In a two-stage laser-induced model, *GzmB*-deficient mice developed smaller collagen I-positive subretinal fibrotic lesions than wild-type mice. Subsequent mechanistic studies implicated GzmB in modulating inflammatory responses and RPE EMT, supporting its contribution to subretinal fibrosis [342]. However, because the available in vivo evidence is based primarily on genetic deficiency, the therapeutic efficacy and safety of pharmacological GzmB inhibition in subretinal fibrosis remain to be established.

Macrophage-derived SPP1 may link inflammation to mechanotransduction by engaging CD44 on neighboring stromal or vascular cells and activating Rho-dependent YAP signaling. The broader RhoA–ROCK–YAP axis promotes actomyosin contractility and profibrotic gene expression, providing a rationale for pharmacological inhibition of ROCK with agents such as ripasudil [203,343]. Ripasudil (Kowa Company, Ltd., Nagoya, Japan) is a small-molecule ROCK inhibitor that has been clinically used or evaluated for anterior-segment ocular conditions, including glaucoma, ocular hypertension, and corneal edema following cataract surgery [344]. However, the effective and sustained delivery of small-molecule anti-fibrotic agents to the posterior segment of the eye remains challenging. To overcome this limitation, novel drug-delivery systems are being explored. For example, encapsulating ripasudil within liposomes (Lipo-Ripa) significantly increases its intravitreal bioavailability. Intravitreal injection of Lipo-Ripa on day 14 post-laser injury after reduction in CNV significantly reduced subretinal fibrosis. In a rabbit PVR model, Lipo-Ripa injection 3 days after injecting RPE cells also prevented PVR progression [328,329].

Whereas the strategies described above primarily target extracellular mediators, receptor signaling, matrix remodeling, or mechanotransduction, emerging research indicates that fibrotic conversion also depends on cell-intrinsic metabolic reprogramming. TGF-β2-induced RPE EMT is accompanied by suppression of mitochondrial oxidative phosphorylation and a compensatory shift toward glycolysis [345]. Consistent with a causal role for mitochondrial dysfunction [346], deficiency of the mitochondrial 2-oxoglutarate carrier (OGC, SLC25A11) impaired mitochondrial respiration, activated the PI3K/AKT-dependent Smad signaling, and exacerbated laser-induced subretinal fibrosis in mice. In contrast, OGC overexpression attenuated EMT in cultured RPE cells [347]. Pharmacological restoration of mitochondrial regulation may therefore offer a complementary strategy. In vitro, the small-molecule PGC-1α activator ZLN005 suppressed TGF-β2-induced expression of α-SMA, CTGF, COL1A1, and other mesenchymal markers and reduced RPE cell migration. However, its antifibrotic efficacy has not yet been established in animal models of nAMD-associated subretinal fibrosis [345].

Collectively, these findings highlight the multifactorial nature of subretinal fibrosis and support the development of therapeutic strategies that target complementary extracellular, cellular, mechanical, and metabolic mechanisms. Most preclinical interventions were initiated before or shortly after disease induction and therefore primarily demonstrate prevention of fibrosis initiation or attenuation of its early progression, rather than reversal of established fibrosis. Nevertheless, the efficacy of selected interventions administered at later stages, including Lipo-Ripa treatment initiated 14 days after laser injury, suggests that ongoing or established fibrotic remodeling may remain therapeutically modifiable. However, definitive evidence of fibrosis reversal will require studies that document the presence and extent of fibrosis before treatment and demonstrate regression from this pretreatment baseline.

## 6. Conclusions

In this review, we discussed fibrosis in nAMD and DR, focusing on the cellular origins of myofibroblasts and the mechanisms underlying ECM deposition and remodeling. We summarized the molecular pathways that drive the angio-fibrotic switch, including key regulators of mesenchymal transition, cellular stress responses, and mechanotransduction. We also reviewed current clinical management strategies and their limitations, as well as emerging antifibrotic approaches under preclinical investigation. Ocular fibrosis is a complex, multicellular process involving extensive crosstalk among multiple signaling pathways. A deeper understanding of these interconnected mechanisms will therefore be essential for developing effective combination or multitargeted therapies to prevent or suppress fibrosis in ocular diseases.

## Figures and Tables

**Table 1:** Drugs in clinical trials for AMD and DR that target angiogenesis.

Name	Brand Name	Company	Phase/Status	Format	Route	Conditions	Reference
CKD-701/EB12-20145P	LucenBS	Chong Kun Dang Pharmaceutical	Phase 3 (NCT04857177)	Anti-VEGF Ab	IVT	nAMD	[267]
HLX04-O		Shanghai Henlius Biotech, Essex Bio-Technology	Phase 3 (NCT05003245, NCT04740671)	Anti-VEGF Ab	IVT	nAMD	[268]
Tarcocimab tedromer, KSI-301,	Zenkuda	Kodiak Sciences	Phase 3 DAYLIGHT (NCT04964089), DAYBREAK (NCT06556368) with nAMD, phase 3 GLOW1, 2 with DR (NCT05066230, NCT06270836), phase 3 GLEAM (NCT04611152) and GLIMMER (NCT04603937) with DME, phase 3 BEACON with RVO (NCT04592419)	Anti-VEGF biopolymer	IVT	nAMD, DR, DME, RVO	[269]
RGX-314, surabgene lomparvovec, ABBV-RGX-314		AbbVie, REGENXBIO	Phase 2b/3 ATMOSPHERE (NCT04704921), ASCENT (NCT05407636), AAVIATE (NCT04514653) for nAMD, phase 2 ALTITUDE (NCT04567550) and ELAAVATE (NCT06942520)	AAV8-anti-VEGF Fab	Subretinal or suprachoroidal		[270]
ADVM-022, ixoberogene soroparvovec, Ixo-vec		Adverum Biotechnologies	Phase 3 ARTEMIS (NCT06856577) and AQUARIUS (NCT07482176)	AAV2.7m8-Aflibercept	IVT	nAMD	[271]
LX102		Innostellar Biotherapeutics	Phase 3 STELLAR (NCT07317934)	rAAV2-VEGF-Trap	Subretinal	nAMD	[272]
IBI333		Innovent Biologics	Phase 1 (NCT05639530)	Bispecific decoy targeting VEGF-A/C	IVT	nAMD	[273]
4D-150		4D Molecular Therapeutics (4DMT)	Phase 3 4 FRONT-1 (NCT06864988) and 4 FRONT-2 (NCT07064759)	AAV capsid Aflibercept and RNAi for VEGF-C	IVT	nAMD	[274]
EXG102-031		Exegenesis Bio	Phase 1 EVEREST (NCT05903794)	rAAV targeting VEGF and ANG2	subretinal	nAMD	[275]
Gersizangitide, AXT107		AsclepiX Therapeutics	Phase 1/2a DISCOVER (NCT05859776) with nAMD	Col IV-derived peptide inhibiting VEGFR2 while activating TIE2	suprachoroidal	nAMD, DME	[276]
OTX-TKI	AXPAXLI	Ocular Therapeutix	Phase 3 SOL-1 (NCT06223958), SOL-R (NCT06495918) SOL-X (NCT07516132) withnAMD, phase 3 HELIOS-3 with NPDR (NCT07235085)	TKI for VEGFR1, VEGFR2, VEGFR3 (axitinib)	Bioresorbable intravitreal hydrogel implant	nAMD, NPDR	[277]
CLS-AX		Clearside Biomedical	Phase 2b ODYSSEY (NCT05891548)	TKI for VEGFR1, VEGFR2, VEGFR3 (axitinib)	suprachoroidal	nAMD	[278]
Lorutengitide, EB-203		EyeBioKorea	Phase 2a (NCT06487039)	Collagen-derived synthetic peptide	eyedrop	nAMD	[279]
BI 764524		Boehringer Ingelheim	Phase 2 CRIMSON (NCT06321302)	Anti-Sema3A Ab	IVT	DR, DMI	[280,281]
DS-7080a		Daiichi Sankyo	Phase 1 (NCT02530918)	Anti-ROBO4	IVT	nAMD, DME	[282]

**Table 2:** Drugs Targeting Fibrosis-Related Pathways for AMD and DR in Clinical Trials.

Name	Brand Name	Company	Phase/Status	Format	Route	Conditions	Reference
Tabirafusp tedromer, KSI-501		Kodiak Sciences	Phase 3 DAYBREAK with nAMD (NCT06556368), phase 3 ALTO with DME (NCT07734844)	Bispecific Ab biopolymer targeting VEGF and IL-6	IVT	nAMD, DME	[289]
eflimrufusp alfa, RC28-E		RemeGen, Santen	Phase 1 (NCT03777254) and phase 3 (NCT05727397) with nAMD, phase 2 (NCT04782115) and phase 3 (NCT05885503) with DME	Bispecific decoy targeting VEGF and FGF-2	IVT	nAMD, DME	[290,291]
Efdamrofusp alfa, IBI302		Innovent Biologics	Phase 2 (NCT04820452, NCT05961007)	bifunctional decoy receptor targeting VEGF and C3b/C4b	IVT	nAMD	[292,293]
EYP-1901	DURAVYU	EyePoint Pharmaceuticals	Phase 3 LUGANO and LUCIA with nAMD (NCT06668064, NCT06683742), phase 3 COMO and CAPRI with DME (NCT07449936, NCT07449923)	TKI for VEGFR, PDGFR (vorolanib)	Bioerodible intravitreal insert	nAMD, DME	[294,295]
Fenofibrate		Fournier Pharma SA	FIELD (ISRCTN#64783481), phase 3 ACCORD-EYE (NCT00542178), phase 4 LENS (NCT03439345)	PPARα agonist	oral	DR	[296,297,298,299]
Canakinumab, ACZ885	ILARIS	Novartis	Phase 1 (NCT00503022)	Anti-IL-1β Ab	IVT	nAMD	[300]
ANX007		Annexon	Phase 2 ARCHER (NCT04656561)	Anti-C1q Ab fragment	IVT	GA	[172,301]
Iptacopan, LNP023	Fabhalta	Novartis	Phase 2 (NCT05230537)	Complement factor B inhibitor	oral	AMD	[302]
AVD-104		Aviceda Therapeutics	Phase 2 with DME (NCT06181227), phase 2a SIGLEC (NCT05839041)	Glycoimmune therapeutic	IVT	GA	
Risuteganib, ALG-1001	Luminate	Allegro Ophthalmics	Phase 2 with dry AMD (NCT03626636), phase 1/2 with AMD (NCT01749891), phase 2 with DME (NCT02348918), phase 2 with vitreomacular adhesion (NCT02153476)	Anti-integrin oligopeptide		Dry AMD, DME	[303]
Brimapitide, XG-102, AM-111, D-JNKI-1		Xigen	Phase 3 (NCT02235272, NCT02508337)	A TAT-coupled peptide inhibitor for JNK	subconjunctival	Postoperative ocular inflammation	[304]

**Table 3:** Preclinical Strategies for Fibrosis.

Name	MOA	Route	Model	Reference
TGF-β NAb	TGF-β neutralizing Ab	i.p.	Subretinal injection of macrophage-rich peritoneal exudate cells in mice	[86]
Fenofibrate	PPARα activator	Fenofibrate-containing chow	*Vldlr*^−/−^ mice	[325]
RO4929097	γ-Secretase Inhibitor	IVT	Single laser-induced CNV and subretinal fibrosis in young and aged mice	[326]
Mudan granules	Fibrillin-2 restoration	intragastric	STZ-induced diabetic mice	[327]
MMP408	MMP12 inhibitor	Gavage	Two-stage laser-induced mouse model	[59]
Liposome-encapsulated ripasudil	ROCK inhibitor	IVT	Single laser-induced CNV in mice and PVR model in rabbits	[328,329]

## Data Availability

No new data were created or analyzed in this study. Data sharing is not applicable to this article.

## Abbreviations

The following abbreviations are used in this manuscript:

AAV	adeno-associated virus
ABC	antibody biopolymer conjugate
ADAM	a disintegrin and metalloprotease
AGEs	advanced glycation end products
aMMP-8	active matrix metalloproteinase-8
ANG2	Angiopoietin 2
α-SMA	α-smooth muscle actin
BCVA	best-corrected visual acuity
BM	basement membrane
BMDMs	bone marrow-derived macrophages
BRB	blood–retina barrier
CCR2	C-C motif chemokine receptor 2
CECs	choroidal endothelial cells
CNV	choroidal neovascularization
COST	cone outer segment termination
CTGF	connective tissue growth factor
CX3CL1	C-X3-C motif chemokine ligand 1
CX3CR1	C-X3-C motif chemokine receptor 1
CXCL8	C-X-C motif chemokine ligand 8
DAF	decay-accelerating factor
DME	diabetic macular edema
DMI	diabetic macular ischemia
DR	diabetic retinopathy
DRIL	disorganization of the retinal inner layers
ECM	extracellular matrix
ELM	external limiting membrane
EMT	epithelial-to-mesenchymal transition
EndMT	endothelial-to-mesenchymal transition
ERM	epiretinal membrane
EZ	ellipsoid zone
FAK	focal adhesion kinase
FBN2	fibrillin-2
FDA	Food and Drug Administration
FH	factor H
FI	factor I
FSP-1	fibroblast-specific protein-1
FVM	fibrovascular membranes
GA	geographic atrophy
GAG	glycosaminoglycans
GALNS	galactosamine-6 sulfatase
GPCR	G protein-coupled receptor
HIF-1α	hypoxia-inducible factor-1α
IL-1β	interleukin-1β
LAP	latency-associated peptide
LATS	large tumor suppressor kinases
MAC	membrane attack complex
MCP	membrane cofactor protein
MCP-1	monocyte chemoattractant protein-1
MLCs	myosin light chains
MLCK	myosin light chain kinase
MMT	macrophage-to-myofibroblast transition
MNV	macular neovascularization
MST	mammalian STE20-like kinases
NAD+	nicotinamide adenine dinucleotide
nAMD	neovascular age-related macular degeneration
NF-κB	nuclear factor-κB
NICD	Notch intracellular domain
NLRP3	the NLR family pyrin domain-containing 3
NPDR	non-proliferative diabetic retinopathy
OCT	optical coherence tomography
OCTA	OCT-angiography
PBMCs	peripheral blood mononuclear cells
PDGF	platelet-derived growth factor
PDR	proliferative diabetic retinopathy
PDS	Port Delivery System
PMT	pericyte-to-myofibroblast transition
PPV	pars plana vitrectomy
RAGE	receptor for AGE
RGD	arginine–glycine–aspartate
ROCK	Rho-associated coiled-coil-containing protein kinase
ROS	reactive oxygen species
RPE	retinal pigment epithelium
S1P	sphingosine-1-phosphate
SHRM	subretinal hyperreflective material
SRF	subretinal fluid
T&E	treat-and-extend
TEAD	TEA domain family of DNA-binding proteins
TGF-β	transforming growth factor beta
TKI	tyrosine kinase inhibitor
TNF	tumor necrosis factor
TRD	tractional retinal detachment
VEGF-A	vascular endothelial growth factor A
ZEB	zinc finger E-box binding homeobox
ZO-1	Zonula Occludens-1
